# vhp Is a Fibrinogen-Binding Protein Related to vWbp in Staphylococcus aureus

**DOI:** 10.1128/mBio.01167-21

**Published:** 2021-08-03

**Authors:** Sheila Thomas, Srishtee Arora, Wen Liu, Kelly Churion, You Wu, Magnus Höök

**Affiliations:** a Center for Infectious and Inflammatory Diseases, Institute of Biosciences and Technology, Texas A&M University Health Science Center, Houston, Texas, USA; St. Jude Children’s Research Hospital

**Keywords:** *Staphylococcus aureus*, fibrinogen-binding proteins, vhp, vWbp homolog

## Abstract

Staphylococcus aureus can target a variety of tissues, causing life-threatening infections. The basis for this diversity stems from the microorganism’s ability to spread in the vascular system throughout the body. To survive in blood, S. aureus coats itself with a fibrinogen (Fg)/fibrin shield. The protective shield is assembled by the coordinated actions of a number of Fg-binding bacterial proteins that manipulate the host’s blood coagulation system. Several of the Fg binders appear redundant, sharing similar functional motifs. This observation led us to screen for the presence of novel proteins with significant amino acid identities to von Willebrand factor-binding protein (vWbp), a key component in the shield assembly machinery. One identified protein showed significant sequence identity with the C-terminal region of vWbp, and we consequently named it vWbp homologous protein (vhp). The *vhp* gene lies within a cluster of genes that encode other virulence factors in S. aureus. Although each isolate only contains one copy of the *vhp* gene, S. aureus has at least three distinct alleles, *vhpA*, *B*, and *C*, that are present in the core genome. All three vhp isoforms bind Fg with high affinity, targeting a site located in the D fragment of Fg. We further identified an ∼79 amino acid-long, conserved segment within the C-terminal region of vWbp that shares high sequence identities (54 to 67%) with the vhps and binds soluble Fg with high affinity. Further analysis of this conserved motif and the intact vhps revealed intriguing differences in the Fg binding behavior, perhaps suggesting that these proteins have similar but discrete functions in the shield assembly.

## INTRODUCTION

The Gram-positive, opportunistic pathogen Staphylococcus aureus employs a multitude of molecular tactics to evade the host defense system. Assembling a protective fibrinogen (Fg)/fibrin shield around the bacterium appears to be particularly important for staphylococcal survival in blood (1–4). The Fg shield protects S. aureus from being engulfed by neutrophils and macrophages (1, 2). The resulting impaired bacterial clearance allows for S. aureus to propagate and disseminate into surrounding tissues, causing serious infections, such as sepsis, osteomyelitis, and endocarditis (5, 6).

Fg is an abundant (2 to 5 mg/ml) plasma glycoprotein that is expressed by hepatocytes in the liver (7–9). It consists of three pairs of nonidentical chains, Aα-, Bβ-, and γ-chains, that are linked by 29 disulfide bonds (8–11). A central domain known as the E region is adjoined to two lateral globular D regions via triple α-helical coil-coiled segments (9, 11). Discrete E and D fragments can be isolated after digestion of full-length Fg by plasmin (8–12).

Although Fg is best known for its role in blood coagulation, it also has an important role in the defense against pathogenic microbes. Upon tissue damage, synthesis of Fg is upregulated and is recruited to the site of injury, where it is converted to insoluble fibrin via the coagulation cascade (12, 13). Fibrin(ogen) adheres to the “platelet plug” to create the fibrin clot, curbing bleeding and impeding bacterial entry (3, 9, 11). In addition, Fg regulates bacterial clearance by neutrophils via its interaction with the leukocyte integrin receptor α_m_β_2_, resulting in neutrophil adhesion to the endothelial surface and the release of signals for activating proinflammatory pathways (3, 14, 15). Given the importance of Fg in the defense against pathogenic microbes, it is no surprise that some bacteria attempt to counter this activity (3, 16–18).

S. aureus exploits the host’s Fg and blood coagulation system in efforts to escape recognition and clearance by the host defense mechanisms. To this end, the microorganism expresses a number of Fg-binding proteins (3, 17–21). These include a group of structurally related cell wall-anchored proteins that belong to the microbial surface components recognizing adhesive matrix molecules (MSCRAMM) family (3, 17, 18) as well as a group of secreted proteins belonging to a protein family referred to as the secretable expanded repertoire adhesive molecules (SERAMs) (3, 6, 17, 19–21). Most of these Fg-binding proteins have been shown to act as virulence factors in different animal models of staphylococcal infections, and, in some cases, the Fg binding activities of these proteins have been shown to be required for their virulence activity (3, 4, 6, 19, 20, 22–24).

Many of the interactions between staphylococcal proteins and Fg have not been characterized in detail. Thus, there is an incomplete picture of how these proteins manipulate the host’s Fg physiology. One of the key S. aureus Fg-binding proteins is von Willebrand factor-binding protein (vWbp), which can act as a coagulase but is also a high-affinity Fg binder (25–29). vWbp has been demonstrated to act as a virulence factor in a murine bacteremia model, although the specific feature or features of vWbp that are responsible for the protein’s pathogenic activity remain unknown (1, 3, 25, 26). vWbp has at least two distinct Fg-binding sites located at its N- and C-terminal regions, respectively (27–29). The N-terminal region of vWbp exhibits a slightly higher affinity to immobilized Fg than its C-terminal half (reported apparent half-maximal binding concentrations [*K_D_*] of 3.2 nM versus 38 nM, respectively [28]) and binds specifically to the N-terminal segment of the Fg β-chain (27). The C-terminal Fg-binding site has not been characterized. Although the C-terminal Fg-binding domain of vWbp is highly conserved among S. aureus isolates, the C-terminal region does not contain any of the known staphylococcal Fg-binding motifs (27, 30–32).

In the current study, we have identified a unique protein, vWbp homologous protein (vhp), that shows a high degree of amino acid sequence identity to a C-terminal domain in vWbp and binds Fg with high affinity. Although each isolate only contains one copy of the *vhp* gene, S. aureus can express at least three distinct isoforms, vhpA, B, and C. This Fg-binding activity of the three vhp isoforms is further characterized.

## RESULTS

### An S. aureus open reading frame encodes a unique protein homolog of the C-terminal section of vWbp.

**FIG 1:**
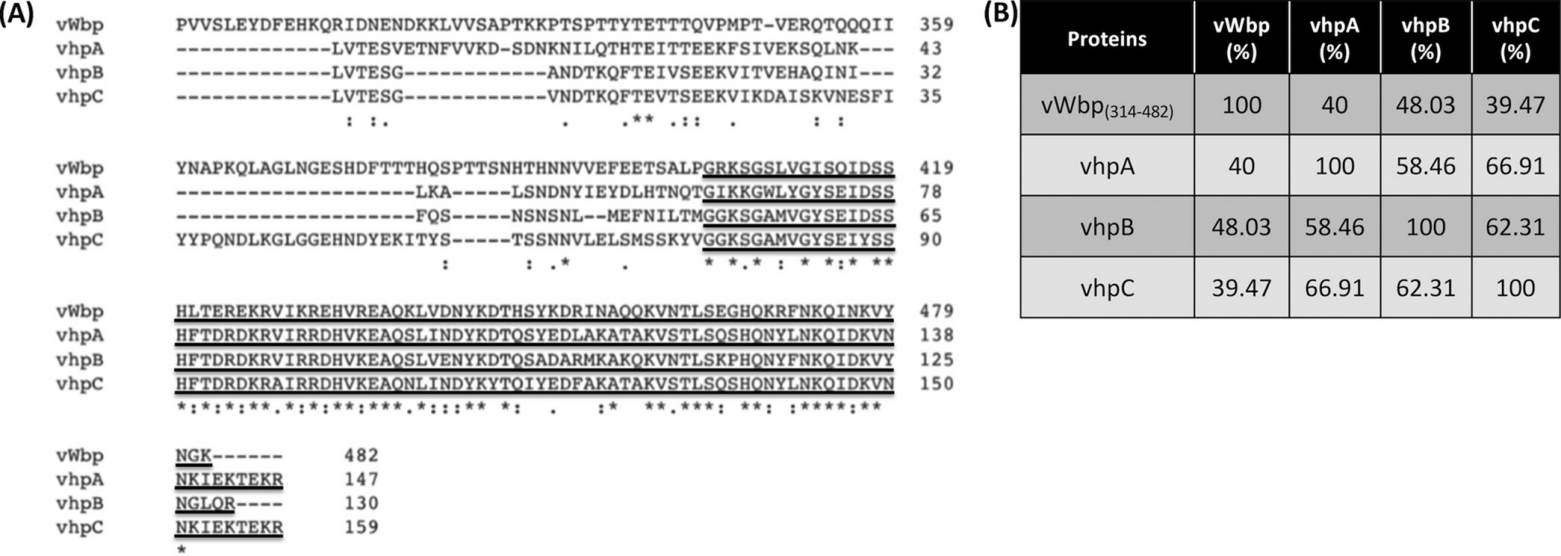
vhp is homologous to vWbp-C_(386 to 482)_. (A) Clustal alignment of C-terminal vWbp, covering residues 242 to 482, and full-length vhpA, vhpB, and vhpC. Underlined residues indicate the conserved region. (B) Percent amino acid identity of vWbp_(314 to 482)_ S. aureus Newman (AAK52333.1), vhpA S. aureus N315 (BAB41978.1), vhpB S. aureus USA300_ FPR3757 (ABD21761.1), and vhpC S. aureus TCH60 (ADQ77850.1).

We initiated a search for proteins homologous to vWbp in S. aureus genomes by using BLAST with the full-length vWbp sequence. Our search identified three related hypothetical proteins that have a signal peptide at the N-terminal region and a relatively conserved segment at their C-terminal region that is shared with the C-terminal section of vWbp (40%, 48%, and 39% identity, respectively) (Fig. 1A and B) and were subsequently determined to be isoforms (see below). We called these proteins vWbp homologous protein A, B, or C (vhpA, vhpB, or vhpC, respectively). Full-length vhpA shares a 58% and 67% amino acid identity with that of full-length vhpB and vhpC, respectively (Fig. 1B). Between vhpB and vhpC there is a 62% sequence identity (Fig. 1B). However, the conserved C-terminal segment of vhpA (residues 63 to 147) shares a 70% identity with vhpB (residues 50 to 130) and an 87% identity with vhpC (residues 75 to 159) (Table S1 in the supplemental material). The percent identity of vhpB (residues 50 to 130) with vhpC (residues 75 to 159) is 70% (Table S1).

10.1128/mBio.01167-21.1 TABLE S1 Percent amino acid identity of conserved C-terminal region of vWbp and vhp. Download Table S1, PDF file, 0.1 MB. Copyright © 2021 Thomas et al. 2021 Thomas et al. https://creativecommons.org/licenses/by/4.0/ This content is distributed under the terms of the Creative Commons Attribution 4.0 International license.

The vhpA, B, and C proteins are smaller than the 508 amino acid residue-long vWbp and are 173, 156, and 185 amino acid residues long, respectively (Fig. 1A and Fig. S1A and B). The vhps lack the two D domains found in the N terminus of staphylocoagulase (Coa) and vWbp, the characteristic first two N-terminal amino acids Val-Val of vWbp (33), and the Ile-Val of Coa (34) required for prothrombin activation. Therefore, vhps likely do not act as coagulases. The vhps also lack the 26 amino acid-long vWF-binding motif that is present in vWbp (Fig. 1 and Fig. S1A) (35).

10.1128/mBio.01167-21.4 FIG S1 Sequence alignments of vhp (A) and vWbp (B) isoforms. Download FIG S1, PDF file, 0.1 MB. Copyright © 2021 Thomas et al. 2021 Thomas et al. https://creativecommons.org/licenses/by/4.0/ This content is distributed under the terms of the Creative Commons Attribution 4.0 International license.

### The *vhp* gene is located in a gene cluster encoding known virulence factors.

**FIG 2:**
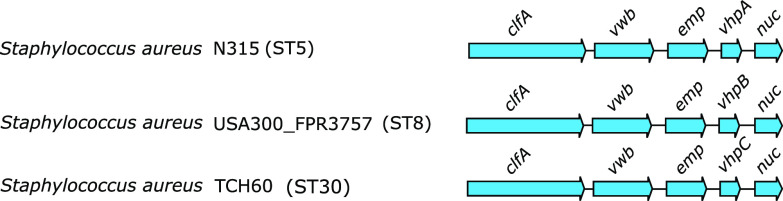
The *vhp* gene is located in a gene cluster encoding known virulence factors. A schematic representation of the organization of genes in the gene cluster. Blue arrowhead indicates a gene and the direction of translation. ST, sequence type.

We located the gene encoding vhpA protein in the S. aureus strain N315 genome to a gene cluster that encodes known virulence factors. The gene cluster consists of *clfA*, *vWb*, *emp*, *vhp*, and *nuc* genes (Fig. 2) (35–37). This location is also observed for the *vhpB* gene from strain USA300_FPR3757 and the *vhpC* gene of strain TCH60 (Fig. 2). Thus, all three *vhp* genes are encoded in the same genetic location in different S. aureus strains, demonstrating that vhpA, B, and C proteins are isoforms of each other.

It is noteworthy that *clfA*, *vWb*, and *emp* genes encode Fg-binding proteins (21, 23, 27) that are known virulence factors, as demonstrated in murine S. aureus infection models (22, 23, 25, 38). Also, nuclease (Nuc) plays a role in virulence by degrading extracellular DNA traps released by neutrophils (37).

### vhp is present in S. aureus as distinct isoform groups.

We analyzed the genome sequences of 30 published clinical isolates with the goal to determine the genetic variation of *vhp* in relation to *vWb*. The isolates were obtained from different types of S. aureus infections from various geographical regions and represent a variety of sequence types (ST) (Table 1). Since the amino acid sequence differences between vhpA, B, and C were substantial, we referred to these sequences as prototypes of specific isotype groups (Table 1). We chose strain N315 as the prototype for vhpA, USA300_FPR3757 for vhpB, and TCH60 for vhpC, as these are also well-established clinical isolates. We assigned vhp isoforms to a particular group if they had an amino acid identity of 85 to 100% compared to the prototype of their respective isotype group (Table 1).

**TABLE 1:** Percent amino acid identity of vhp in 30 clinical S. aureus isolates

Strain name	ST^*a*^	vhp isoforms^*b*^	Protein ID^*c*^	CAI80 435.1^*d*^	ANI73 772.1^*d*^	AGO2 9166.1^*d*^	ADL2 2693.1^*d*^	CCG1 5434.1^*d*^	ADI97 346.1^*d*^	BAB9 4633.1^*d*^	AGU6 0934.1^*d*^	AMV8 4570.1^*d*^	BAB4 1978.1^*d*^	BAF7 7693.1^*d*^	ABR5 1687.1^*d*^	ADC3 6980.1^*d*^	AQD1 8902.1^*d*^	CCJ10 585.1^*d*^	CAG3 9854.1^*d*^	ADQ7 7850.1^*d*^	AIO20 462.1^*d*^	CRL3 3498.1^*d*^	AFH6 9102.1^*d*^	ABD2 9944.1^*d*^	AEW6 4856.1^*d*^	ABD2 1761.1^*d*^	AAW3 6414.1^*d*^	ELP41 198.1^*d*^	EJE56 573.1^*d*^	CBI48 744.1^*d*^	AEV7 7849.1^*d*^	ALQ9 9050.1^*d*^	AGU5 4604.1^*d*^
RF122	151	vhpA'	CAI80435.1	100^*e*^	96.45^*e*^	83.43^*e*^	85.8^*e*^	84.02^*e*^	86.39^*e*^	84.62^*e*^	86.39^*e*^	86.39^*e*^	86.98^*e*^	86.98^*e*^	86.98^*e*^	86.98^*e*^	86.98^*e*^	85.8^*e*^	56.96	56.96	56.96	61.39	60.9	78.21	78.21	78.21	78.21	78.21	76.87	78.21	77.56	77.56	67.86
08-02300	7	vhpA'	ANI73772.1	96.45^*e*^	100^*e*^	83.43^*e*^	84.62^*e*^	84.02^*e*^	86.39^*e*^	84.62^*e*^	86.39^*e*^	86.39^*e*^	88.17^*e*^	88.17^*e*^	88.17^*e*^	88.17^*e*^	88.17^*e*^	86.98^*e*^	55.7	55.7	55.7	60.13	59.62	75.64	75.64	75.64	75.64	75.64	74.15	75.64	75	75	66.07
CA-347	45	vhpA	AGO29166.1	83.43^*e*^	83.43^*e*^	100^*e*^	93.64^*e*^	93.06^*e*^	93.64^*e*^	94.8^*e*^	94.22^*e*^	94.22^*e*^	93.64^*e*^	93.64^*e*^	93.64^*e*^	93.64^*e*^	93.64^*e*^	92.49^*e*^	70.37	70.37	70.37	73.46	70.62	63.46	63.46	63.46	63.46	63.46	61.22	63.46	62.82	62.82	66.07
JKD61 59	93	vhpA	ADL22693.1	85.8^*e*^	84.62^*e*^	93.64^*e*^	100^*e*^	92.49^*e*^	94.8^*e*^	95.38^*e*^	94.8^*e*^	94.8^*e*^	95.38^*e*^	95.38^*e*^	95.38^*e*^	95.38^*e*^	95.38^*e*^	94.22^*e*^	69.14	69.14	69.14	72.84	70.62	63.46	63.46	63.46	63.46	63.46	61.22	63.46	62.82	62.82	66.96
HO 5096 0412	22	vhpA	CCG15434.1	84.02^*e*^	84.02^*e*^	93.06^*e*^	92.49^*e*^	100^*e*^	95.95^*e*^	94.8^*e*^	96.53^*e*^	96.53^*e*^	95.95^*e*^	95.95^*e*^	95.95^*e*^	95.95^*e*^	95.95^*e*^	94.8^*e*^	69.75	69.75	69.75	72.22	74.38	64.1	64.1	64.1	64.1	64.1	61.9	64.1	63.46	63.46	65.18
ED133	133	vhpA	ADI97346.1	86.39^*e*^	86.39^*e*^	93.64^*e*^	94.8^*e*^	95.95^*e*^	100^*e*^	95.95^*e*^	97.69^*e*^	97.69^*e*^	98.27^*e*^	98.27^*e*^	98.27^*e*^	98.27^*e*^	98.27^*e*^	97.11^*e*^	69.14	69.14	69.14	74.07	73.75	66.03	66.03	66.03	66.03	66.03	63.95	66.03	65.38	65.38	67.86
MW2	1	vhpA	BAB94633.1	84.62^*e*^	84.62^*e*^	94.8^*e*^	95.38^*e*^	94.8^*e*^	95.95^*e*^	100^*e*^	98.27^*e*^	98.27^*e*^	96.53^*e*^	96.53^*e*^	96.53^*e*^	96.53^*e*^	96.53^*e*^	95.38^*e*^	69.14	69.14	69.14	74.69	72.5	64.1	64.1	64.1	64.1	64.1	61.9	64.1	63.46	63.46	66.96
CN1	72	vhpA	AGU60934.1	86.39^*e*^	86.39^*e*^	94.22^*e*^	94.8^*e*^	96.53^*e*^	97.69^*e*^	98.27^*e*^	100^*e*^	100^*e*^	98.27^*e*^	98.27^*e*^	98.27^*e*^	98.27^*e*^	98.27^*e*^	97.11^*e*^	69.14	69.14	69.14	74.07	73.75	66.03	66.03	66.03	66.03	66.03	63.95	66.03	65.38	65.38	67.86
ST201 30943	25	vhpA	AMV84570.1	86.39^*e*^	86.39^*e*^	94.22^*e*^	94.8^*e*^	96.53^*e*^	97.69^*e*^	98.27^*e*^	100^*e*^	100^*e*^	98.27^*e*^	98.27^*e*^	98.27^*e*^	98.27^*e*^	98.27^*e*^	97.11^*e*^	69.14	69.14	69.14	74.07	73.75	66.03	66.03	66.03	66.03	66.03	63.95	66.03	65.38	65.38	67.86
N315^*f*^	5	vhpA	BAB41978.1	86.98^*e*^	88.17^*e*^	93.64^*e*^	95.38^*e*^	95.95^*e*^	98.27^*e*^	96.53^*e*^	98.27^*e*^	98.27^*e*^	100^*e*^	100^*e*^	100^*e*^	100^*e*^	100^*e*^	98.84^*e*^	68.52	68.52	68.52	73.46	73.12	65.38	65.38	65.38	65.38	65.38	63.27	65.38	64.74	64.74	66.96
Mu3	5	vhpA	BAF77693.1	86.98^*e*^	88.17^*e*^	93.64^*e*^	95.38^*e*^	95.95^*e*^	98.27^*e*^	96.53^*e*^	98.27^*e*^	98.27^*e*^	100^*e*^	100^*e*^	100^*e*^	100^*e*^	100^*e*^	98.84^*e*^	68.52	68.52	68.52	73.46	73.12	65.38	65.38	65.38	65.38	65.38	63.27	65.38	64.74	64.74	66.96
JH1	105	vhpA	ABR51687.1	86.98^*e*^	88.17^*e*^	93.64^*e*^	95.38^*e*^	95.95^*e*^	98.27^*e*^	96.53^*e*^	98.27^*e*^	98.27^*e*^	100^*e*^	100^*e*^	100^*e*^	100^*e*^	100^*e*^	98.84^*e*^	68.52	68.52	68.52	73.46	73.12	65.38	65.38	65.38	65.38	65.38	63.27	65.38	64.74	64.74	66.96
04-02981	225	vhpA	ADC36980.1	86.98^*e*^	88.17^*e*^	93.64^*e*^	95.38^*e*^	95.95^*e*^	98.27^*e*^	96.53^*e*^	98.27^*e*^	98.27^*e*^	100^*e*^	100^*e*^	100^*e*^	100^*e*^	100^*e*^	98.84^*e*^	68.52	68.52	68.52	73.46	73.12	65.38	65.38	65.38	65.38	65.38	63.27	65.38	64.74	64.74	66.96
ST88	88	vhpA	AQD18902.1	86.98^*e*^	88.17^*e*^	93.64^*e*^	95.38^*e*^	95.95^*e*^	98.27^*e*^	96.53^*e*^	98.27^*e*^	98.27^*e*^	100^*e*^	100^*e*^	100^*e*^	100^*e*^	100^*e*^	98.84^*e*^	68.52	68.52	68.52	73.46	73.12	65.38	65.38	65.38	65.38	65.38	63.27	65.38	64.74	64.74	66.96
ST228	228	vhpA	CCJ10585.1	85.8^*e*^	86.98^*e*^	92.49^*e*^	94.22^*e*^	94.8^*e*^	97.11^*e*^	95.38^*e*^	97.11^*e*^	97.11^*e*^	98.84^*e*^	98.84^*e*^	98.84^*e*^	98.84^*e*^	98.84^*e*^	100^*e*^	67.9	67.9	67.9	72.22	71.88	64.1	64.1	64.1	64.1	64.1	61.9	64.1	63.46	63.46	65.18
MRSA 252	36	vhpC	CAG39854.1	56.96	55.7	70.37	69.14	69.75	69.14	69.14	69.14	69.14	68.52	68.52	68.52	68.52	68.52	67.9	100^*h*^	100^*h*^	100^*h*^	90.81^*h*^	83.12	66.67	66.67	66.67	66.67	66.67	64.63	66.67	64.74	64.74	68.75
TCH60^*g*^	30	vhpC	ADQ77850.1	56.96	55.7	70.37	69.14	69.75	69.14	69.14	69.14	69.14	68.52	68.52	68.52	68.52	68.52	67.9	100^*h*^	100^*h*^	100^*h*^	90.81^*h*^	83.12	66.67	66.67	66.67	66.67	66.67	64.63	66.67	64.74	64.74	68.75
ATCC 25923	243	vhpC	AIO20462.1	56.96	55.7	70.37	69.14	69.75	69.14	69.14	69.14	69.14	68.52	68.52	68.52	68.52	68.52	67.9	100^*h*^	100^*h*^	100^*h*^	90.81^*h*^	83.12	66.67	66.67	66.67	66.67	66.67	64.63	66.67	64.74	64.74	68.75
BB155	152	vhpC'	CRL33498.1	61.39	60.13	73.46	72.84	72.22	74.07	74.69	74.07	74.07	73.46	73.46	73.46	73.46	73.46	72.22	90.81^*h*^	90.81^*h*^	90.81^*h*^	100^*h*^	77.5	71.79	71.79	71.79	71.79	71.79	70.07	71.79	69.87	69.87	75.89
71193	398	vhpB'	AFH69102.1	60.9	59.62	70.62	70.62	74.38	73.75	72.5	73.75	73.75	73.12	73.12	73.12	73.12	73.12	71.88	83.12	83.12	83.12	77.5	100^*i*^	79.49^*i*^	79.49^*i*^	79.49^*i*^	79.49^*i*^	79.49^*i*^	78.91^*i*^	79.49^*i*^	78.21^*i*^	78.21^*i*^	85.71^*i*^
NTCT 8325	8	vhpB	ABD29944.1	78.21	75.64	63.46	63.46	64.1	66.03	64.1	66.03	66.03	65.38	65.38	65.38	65.38	65.38	64.1	66.67	66.67	66.67	71.79	79.49^*i*^	100^*j*^	100^*j*^	100^*j*^	100^*j*^	100^*j*^	100^*j*^	100^*j*^	97.44^*j*^	97.44^*j*^	95.54^*j*^
11819-97	80	vhpB	AEW64856.1	78.21	75.64	63.46	63.46	64.1	66.03	64.1	66.03	66.03	65.38	65.38	65.38	65.38	65.38	64.1	66.67	66.67	66.67	71.79	79.49^*i*^	100^*j*^	100^*j*^	100^*j*^	100^*j*^	100^*j*^	100^*j*^	100^*j*^	97.44^*j*^	97.44^*j*^	95.54^*j*^
USA300_FP R3757^*k*^	8	vhpB	ABD21761.1	78.21	75.64	63.46	63.46	64.1	66.03	64.1	66.03	66.03	65.38	65.38	65.38	65.38	65.38	64.1	66.67	66.67	66.67	71.79	79.49^*i*^	100^*j*^	100^*j*^	100^*j*^	100^*j*^	100^*j*^	100^*j*^	100^*j*^	97.44^*j*^	97.44^*j*^	95.54^*j*^
COL	250	vhpB	AAW36414.1	78.21	75.64	63.46	63.46	64.1	66.03	64.1	66.03	66.03	65.38	65.38	65.38	65.38	65.38	64.1	66.67	66.67	66.67	71.79	79.49^*i*^	100^*j*^	100^*j*^	100^*j*^	100^*j*^	100^*j*^	100^*j*^	100^*j*^	97.44^*j*^	97.44^*j*^	95.54^*j*^
21282	254	vhpB	ELP41198.1	78.21	75.64	63.46	63.46	64.1	66.03	64.1	66.03	66.03	65.38	65.38	65.38	65.38	65.38	64.1	66.67	66.67	66.67	71.79	79.49^*i*^	100^*j*^	100^*j*^	100^*j*^	100^*j*^	100^*j*^	100^*j*^	100^*j*^	97.44^*j*^	97.44^*j*^	95.54^*j*^
Newbould 305	115	vhpB	EJE56573.1	76.87	74.15	61.22	61.22	61.9	63.95	61.9	63.95	63.95	63.27	63.27	63.27	63.27	63.27	61.9	64.63	64.63	64.63	70.07	78.91^*i*^	100^*j*^	100^*j*^	100^*j*^	100^*j*^	100^*j*^	100^*j*^	100^*j*^	97.28^*j*^	97.28^*j*^	95.15^*j*^
TW20	239	vhpB	CBI48744.1	78.21	75.64	63.46	63.46	64.1	66.03	64.1	66.03	66.03	65.38	65.38	65.38	65.38	65.38	64.1	66.67	66.67	66.67	71.79	79.49^*i*^	100^*j*^	100^*j*^	100^*j*^	100^*j*^	100^*j*^	100^*j*^	100^*j*^	97.44^*j*^	97.44^*j*^	95.54^*j*^
M013	59	vhpB	AEV77849.1	77.56	75	62.82	62.82	63.46	65.38	63.46	65.38	65.38	64.74	64.74	64.74	64.74	64.74	63.46	64.74	64.74	64.74	69.87	78.21^*i*^	97.44^*j*^	97.44^*j*^	97.44^*j*^	97.44^*j*^	97.44^*j*^	97.28^*j*^	97.44^*j*^	100^*j*^	100^*j*^	99.11^*j*^
MS4	338	vhpB	ALQ99050.1	77.56	75	62.82	62.82	63.46	65.38	63.46	65.38	65.38	64.74	64.74	64.74	64.74	64.74	63.46	64.74	64.74	64.74	69.87	78.21^*i*^	97.44^*j*^	97.44^*j*^	97.44^*j*^	97.44^*j*^	97.44^*j*^	97.28^*j*^	97.44^*j*^	100^*j*^	100^*j*^	99.11^*j*^
6850	50	vhpB	AGU54604.1	67.86	66.07	66.07	66.96	65.18	67.86	66.96	67.86	67.86	66.96	66.96	66.96	66.96	66.96	65.18	68.75	68.75	68.75	75.89	85.71^*i*^	95.54^*j*^	95.54^*j*^	95.54^*j*^	95.54^*j*^	95.54^*j*^	95.15^*j*^	95.54^*j*^	99.11^*j*^	99.11^*j*^	100^*j*^

a ST, sequence type.

b vhp isoforms indicate names of the isoforms.

c Protein ID is the accession number of the vhp isoforms.

d Percent identities of the vhp isoforms.

e vhpA isotype groups.

f Prototype for vhpA isotype group.

g Prototype for vhpC isotype group.

h vhpC isotype groups.

i Hybrid vhpB' isotype groups.

j vhpB isotype groups.

k Prototype for vhpB isotype group.

A *vhp* gene is present in all isolates examined, and a majority (26 of 30) of the isolates examined belong to one of the identified isotype groups (vhpA, B, or C) (Table 1). Four of the 30 isolates, which included strains RF122, 08-02300, BB155, and 71193, do not fall into either group, suggesting the existence of additional isoforms besides vhpA, B, and C. The amino acid sequence from strain 71193 appears as a hybrid of vhpB and vhpC, in which the N-terminal region shows a relatively high similarity to the N-terminal region of vhpB, while its C-terminal region is nearly identical to the C-terminal segment of vhpC (Fig. S1A). Although the N-terminal amino acid sequence for both vhpAs of strains RF122 and 08–02300 are identical to vhpA, the C-terminal regions are identical to that of vhpB (Fig. S1A). Genome analysis of the strains revealed that only one *vhp* gene exists in each isolate.

We further examined a limited data set composed of 41 S. aureus ST5 isolates, 66 ST8 isolates, and 26 ST30 isolates. We found that all ST5 isolates contained vhpA, all ST30 isolates contained vhpC, and 66 of 68 ST8 isolates contained the vhpB isoform (Table S2).

10.1128/mBio.01167-21.2 TABLE S2 vhp isoforms in S. aureus ST5, ST8, and ST30 isolates. Download Table S2, XLSX file, 0.01 MB. Copyright © 2021 Thomas et al. 2021 Thomas et al. https://creativecommons.org/licenses/by/4.0/ This content is distributed under the terms of the Creative Commons Attribution 4.0 International license.

We also analyzed the *vWb* gene to determine if a particular vWbp isoform is associated with any of the discrete vhp isoforms. Analysis of the 30 prototypic strains revealed at least 5 isoforms, which we termed as vWbpA, B, C, D, or E, respectively, based on an amino acid identity of 80 to 100% (Table 2; Table S3; Fig. S1B). The isolates also appear to carry only one *vwb* gene (Table 2) (35). We then examined whether there was a correlation between the vhp and vWbp isoforms (Table 2). It is noteworthy that the vWbpC isoforms are present in the strains that also harbor the vhpA isoforms, and the vWbpD isoforms are found in the strains that have vhpB (Table 2). However, vWbpA, B, and E isoforms do not appear to be present in isolates harboring specific vhp isoforms. Examining a larger set of isolates would likely reveal additional isoforms of both vhp and vWbp. Based on our results, it is possible that the two S. aureus proteins vhp and vWbp have evolved independently of each other.

**TABLE 2:** Percent amino acid identity of vWbp in clinical isolates

Strain name	ST^*a*^	vhp isoforms^*b*^	vWbp isoforms^*c*^	Protein ID (vWbp)^*d*^	AFH69 100.1^*e*^	AMV8 4568.1^*e*^	CRL3 3493.1^*e*^	ANI73 770.1^*e*^	CAI80 433.1^*e*^	EJE56 571.1^*e*^	CCG1 5432.1^*e*^	AEV7 7847.2^*e*^	ALQ9 9048.1^*e*^	ADI97 344.1^*e*^	BAF7 7691.1^*e*^	ABR5 1685.1^*e*^	A DC3 6978.1^*e*^	CCJ10 583.1^*e*^	ABD2 0992.1^*e*^	AAW3 6412.1^*e*^	ELP41 199.1^*e*^	CBI48 742.1^*e*^	AGO2 9164.1^*e*^	ADQ7 7852.1^*e*^	AIO20 460.1^*e*^	AEW6 4854.1^*e*^	ADL2 2691.1^*e*^	AGU5 4602.1^*e*^
RF122	151	vhpA'	vWbpB	CAI80433.1	79.16	82.16	82.93	84.37	100^*f*^	97.19^*f*^	92.59^*f*^	92.18^*f*^	92.18^*f*^	66.19	66.73	66.73	66.73	66.73	66.06	66.06	66.06	66.06	64.04	64.08	64.08	64.53	68.15	67.95
08-02300	7	vhpA'	vWbpA'	ANI73770.1	82.27^*g*^	84.06^*g*^	82.77^*g*^	100^*g*^	84.37	84.97	80.76	83.97	83.97	65.93	68.48	68.69	68.69	68.69	66.27	66.27	66.27	66.27	67.07	66.06	66.06	67.53	67.34	67.54
CA-347	45	vhpA	vWbpE	AGO29164.1	66.8	67.67	66.06	67.07	64.04	65.05	63.98	65.39	65.39	76.25	73.44	73.24	73.24	73.24	76.88	76.88	76.88	76.88	100^*h*^	91.42^*h*^	91.42^*h*^	92.14^*h*^	77.45	77.53
JKD6159	93	vhpA		ADL22691.1	67.27	67.74	65.92	67.34	68.15	67.55	67.07	68.69	68.69	71.8	70.77	70.77	70.77	70.77	73.36	73.36	73.36	73.36	77.45	75.2	75.2	74.21	100	81.15
HO 50960412	22	vhpA	vWbpB	CCG15432.1	79.16	79.76	80.92	80.76	92.59^*f*^	94.19^*f*^	100^*f*^	94.82^*f*^	94.82^*f*^	65.32	65.86	65.86	65.86	65.86	65.19	65.19	65.19	65.19	63.98	65.24	65.24	63.27	67.07	67.88
ED133	133	vhpA	vWbpC'	ADI97344.1	65.26	65.73	64.24	65.93	66.19	66.19	65.32	65.73	65.73	100	84.97^*i*^	84.97^*i*^	84.97^*i*^	84.97^*i*^	71.51	71.51	71.51	71.51	76.25	73.75	73.75	75	71.8	71.6
MW2	1	vhpA	trunc																									
CN1	72	vhpA	trunc																									
ST20130943	25	vhpA	vWbpA	AMV84568.1	93.82^*g*^	100^*g*^	94.39^*g*^	84.06^*g*^	82.16	83.37	79.76	80.36	80.36	65.73	67.68	67.47	67.47	67.47	68.88	68.88	68.88	68.88	67.67	66.06	66.06	66.93	67.74	68.15
N315^*k*^	5	vhpA																										
Mu3	5	vhpA	vWbpC	BAF77691.1	66.4	67.68	66.8	68.48	66.73	67.14	65.86	65.66	65.66	84.97	100^*i*^	99.8^*i*^	99.8^*i*^	99.8^*i*^	70.88	70.88	70.88	70.88	73.44	73.13	73.13	74.2	70.77	71.37
JH1	105	vhpA	vWbpC	ABR51685.1	66.2	67.47	66.6	68.69	66.73	67.14	65.86	65.66	65.66	84.97	99.8^*i*^	100^*i*^	100^*i*^	100^*i*^	71.08	71.08	71.08	71.08	73.24	72.93	72.93	74	70.77	71.37
04-02981	225	vhpA	vWbpC	ADC36978.1	66.2	67.47	66.6	68.69	66.73	67.14	65.86	65.66	65.66	84.97	99.8^*i*^	100^*i*^	100^*i*^	100^*i*^	71.08	71.08	71.08	71.08	73.24	72.93	72.93	74	70.77	71.37
ST88	88	vhpA																										
ST228	228	vhpA	vWbpC	CCJ10583.1	66.2	67.47	66.6	68.69	66.73	67.14	65.86	65.66	65.66	84.97	99.8^*i*^	100^*i*^	100^*i*^	100^*i*^	71.08	71.08	71.08	71.08	73.24	72.93	72.93	74	70.77	71.37
MRSA252	36	vhpC																										
TCH60^*l*^	30	vhpC	vWbpE	ADQ77852.1	68.22	66.06	66.8	66.06	64.08	63.67	65.24	64.43	64.43	73.75	73.13	72.93	72.93	72.93	75.45	75.45	75.45	75.45	91.42^*h*^	100^*h*^	100^*h*^	92.43^*h*^	75.2	78.51
ATCC25923	243	vhpC	vWbpE	AIO20460.1	68.22	66.06	66.8	66.06	64.08	63.67	65.24	64.43	64.43	73.75	73.13	72.93	72.93	72.93	75.45	75.45	75.45	75.45	91.42^*h*^	100^*h*^	100^*h*^	92.43^*h*^	75.2	78.51
BB155	152	vhpC'	vWbpA	CRL33493.1	93.99^*g*^	94.39^*g*^	100^*g*^	82.77^*g*^	82.93	83.53	80.92	80.72	80.72	64.24	66.8	66.6	66.6	66.6	66.46	66.46	66.46	66.46	66.06	66.8	66.8	65.93	65.92	66.94
71193	398	vhpB'	vWbpA	AFH69100.1	100^*g*^	93.82^*g*^	93.99^*g*^	82.27^*g*^	79.16	80.36	79.16	78.96	78.96	65.26	66.40	66.20	66.20	66.20	66.00	66.00	66.00	66.00	66.80	68.22	68.22	65.87	67.27	66.67
NTCT8325	8	vhpB																										
11819-97	80	vhpB	vWbpE	AEW64854.1	65.87	66.93	65.93	67.53	64.53	64.93	63.27	64.87	64.87	75	74.2	74	74	74	76.18	76.18	76.18	76.18	92.14^*h*^	92.43^*h*^	92.43^*h*^	100^*h*^	74.21	76.28
USA300_FPR3757^*m*^	8	vhpB	vWbpD	ABD20992.1	66	68.88	66.46	66.27	66.06	65.66	65.19	65.19	65.19	71.51	70.88	71.08	71.08	71.08	100^*j*^	100^*j*^	100^*j*^	100^*j*^	76.88	75.45	75.45	76.18	73.36	73.07
COL	250	vhpB	vWbpD	AAW36412.1	66	68.88	66.46	66.27	66.06	65.66	65.19	65.19	65.19	71.51	70.88	71.08	71.08	71.08	100^*j*^	100^*j*^	100^*j*^	100^*j*^	76.88	75.45	75.45	76.18	73.36	73.07
21282	254	vhpB	vWbpD	ELP41199.1	66	68.88	66.46	66.27	66.06	65.66	65.19	65.19	65.19	71.51	70.88	71.08	71.08	71.08	100^*j*^	100^*j*^	100^*j*^	100^*j*^	76.88	75.45	75.45	76.18	73.36	73.07
Newbould 305	115	vhpB	vWbpB	EJE56571.1	80.36	83.37	83.53	84.97	97.19^*f*^	100^*f*^	94.19^*f*^	92.18^*f*^	92.18^*f*^	66.19	67.14	67.14	67.14	67.14	65.66	65.66	65.66	65.66	65.05	63.67	63.67	64.93	67.55	67.75
TW20	239	vhpB	vWbpD	CBI48742.1	66	68.88	66.46	66.27	66.06	65.66	65.19	65.19	65.19	71.51	70.88	71.08	71.08	71.08	100^*j*^	100^*j*^	100^*j*^	100^*j*^	76.88	75.45	75.45	76.18	73.36	73.07
M013	59	vhpB	vWbpB	AEV77847.2	78.96	80.36	80.72	83.97	92.18^*f*^	92.18^*f*^	94.82^*f*^	100^*f*^	100^*f*^	65.73	65.66	65.66	65.66	65.66	65.19	65.19	65.19	65.19	65.39	64.43	64.43	64.87	68.69	68.08
MS4	338	vhpB	vWbpB	ALQ99048.1	78.96	80.36	80.72	83.97	92.18^*f*^	92.18^*f*^	94.82^*f*^	100^*f*^	100^*f*^	65.73	65.66	65.66	65.66	65.66	65.19	65.19	65.19	65.19	65.39	64.43	64.43	64.87	68.69	68.08
6850	50	vhpB		AGU54602.1	66.67	68.15	66.94	67.54	67.95	67.75	67.88	68.08	68.08	71.6	71.37	71.37	71.37	71.37	73.07	73.07	73.07	73.07	77.53	78.51	78.51	76.28	81.15	100

a ST, sequence type.

b vhp isoforms indicate names of the isoforms.

c vWbp isoforms indicate names of the isoforms.

d Protein ID is the accession numbers of the vWbp isoforms.

e Percent identities of the vWbp isoforms.

f vWbpB isotype group.

g vWbpA isotype group.

h vWbpE isotype group.

i vWbpC isotype group.

j vWbpD isotype group.

k Prototype for vhpA isotype group.

l Prototype for vhpC isotype group.

m Prototype for vhpB isotype group.

10.1128/mBio.01167-21.3 TABLE S3 Percent amino acid identity of vWbp in clinical S. aureus isolates. Download Table S3, XLSX file, 0.04 MB. Copyright © 2021 Thomas et al. 2021 Thomas et al. https://creativecommons.org/licenses/by/4.0/ This content is distributed under the terms of the Creative Commons Attribution 4.0 International license.

### Recombinant vhpA, B, and C bind Fg with high affinity.

**FIG 3:**
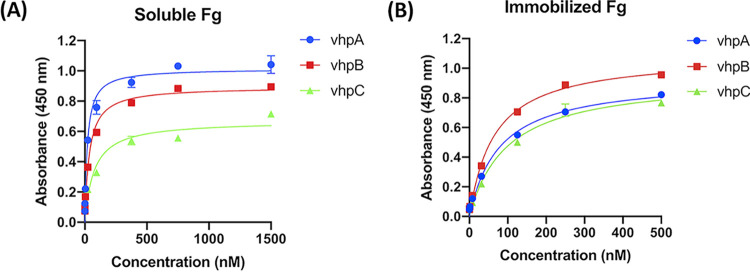
The vhp isoforms bind to immobilized and soluble Fg. (A) ELISA binding of soluble Fg to immobilized vhp isoforms (0.5 μg/well). (B) ELISA binding of vhp isoforms to immobilized Fg (0.5 μg/well). Error bars represent standard error of the mean (SEM). The graphs are representative of three independent experiments.

The C-terminal region of vWbp contains an Fg-binding site (27, 29). We therefore examined the Fg binding activity of the three major vhp isoforms using enzyme-linked immunosorbent assay (ELISA)-type binding assays (Fig. 3A and B) (27). We expressed and purified recombinant full-length vhpA, B, and C, each with a His_6_-maltose-binding protein (MBP) tag fused to the N terminus (Fig. S2A). All three isoforms, when coated in microtiter wells, showed dose-dependent binding to soluble Fg with interactions that exhibited saturation kinetics. The calculated apparent *K_D_* values (concentration required for half-maximum binding) were similar (29 nM, 41 nM, and 87 nM for vhpA, B, and C, respectively [Fig. 3A and Table 3]).

**TABLE 3:** Half-maximal binding concentrations (*K_D_*)

Protein	Soluble Fg (M)^*a*^	Immobilized Fg (M)^*a*^	Immobilized Fg-D (M)^*a*^	Immobilized Fg-E (M)^*a*^
vhpA^*b*^	2.9 × 10^−8^ ± 1.5 × 10^−8^	6.7 × 10 ^−8^ ± 1.0 × 10^−8^	8.10 × 10^−7^ ± 4.3 × 10^−7^	
vhpB^*b*^	4.1 × 10^−8^ ± 2.2 × 10^−8^	5.0 × 10 ^−8^ ± 1.6 × 10^−8^	2.72 × 10^−7^ ± 6.9 × 10^−8^	
vhpC^*b*^	8.7 × 10^−8^ ± 1.2 × 10^−8^	1.0 × 10 ^−7^ ± 4.5 × 10^−8^	2.44 × 10^−7^ ± 7.5 × 10^−8^	
vWbp-C	1.5 × 10^−9^ ± 4.0 × 10^−10^	ND^*c*^	ND	ND
vWbp-C_(386 to 482)_	2.4 × 10^−9^ ± 6.3 × 10^−10^	ND	ND	ND

a Averages of the half-maximal binding concentrations (M) and standard deviations.

b Full-length vhpA, B, and C.

c ND, not determined.

10.1128/mBio.01167-21.5 FIG S2 Both vWbp-C_(386 to 482)_ and vhp bind Fg. (A) SDS-PAGE gel of vhp isoforms and vWbp-C constructs. (B) ELISA binding of soluble Fg to immobilized vhp isoforms (0.5 μg/well). (C) ELISA binding of vhp isoforms to immobilized Fg (0.5 μg/well). (D) ELISA binding of soluble Fg to immobilized vWbp-C constructs (0.5 μg/well). (E) ELISA binding of vWbp-C constructs to immobilized Fg (0.5 μg/well). Error bars represent standard error of the mean (SEM). The graphs are representative of three independent experiments. Download FIG S2, PDF file, 0.8 MB. Copyright © 2021 Thomas et al. 2021 Thomas et al. https://creativecommons.org/licenses/by/4.0/ This content is distributed under the terms of the Creative Commons Attribution 4.0 International license.

When tested for binding to Fg coated on the ELISA plates, the vhp isoforms also bound to immobilized Fg in a dose-dependent manner with apparent *K_D_* values of 67 nM, 50 nM, and 100 nM, respectively (Fig. 3B and Table 3). As a negative control, a recombinant His_6_-MBP fusion protein, purified by the same method used for purifying the recombinant vhps, showed no binding to soluble or immobilized Fg (Fig. S2B and C). Together, our data show that all three isoforms specifically bind to both soluble and immobilized Fg with similar high affinities.

### The vhp isoforms bind to the Fg D fragment.

**FIG 4:**
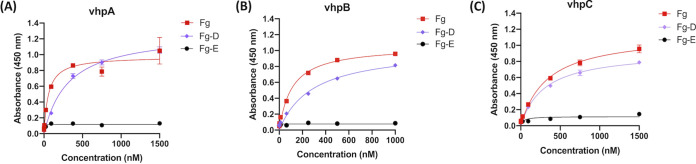
The vhp isoforms bind to the D fragment of Fg. ELISA binding of immobilized Fg fragments (0.5 μg/well) to vhp isoforms vhpA (A), vhpB (B), and vhpC (C); red, full-length Fg; purple, Fg D fragment; black, Fg E fragment. Error bars represent standard error of the mean (SEM). The graphs are representative of three independent experiments.

To further characterize the interaction between Fg and vhp, we sought to identify the segment in Fg that binds to the vhps (Fig. 4A to C). Fg was digested with the fibrinolytic enzyme plasmin to obtain two lateral globular D fragments (Fg-D) and a central E fragment (Fg-E) (7–13). We coated microtiter wells with full-length Fg, Fg-D, or Fg-E and examined the dose-dependent binding of the vhp isoforms to the isolated Fg fragments (Fig. 4A to C). The vhp isoforms bound to immobilized Fg-D in a dose-dependent manner that showed saturation kinetics and showed no binding to immobilized Fg-E. Binding of vhp isoforms to full-length Fg was used as a positive control (Table 3). The calculated apparent *K_D_* values for vhpA, B, and C binding to the Fg-D fragment were 810 nM, 272 nM, and 244 nM, respectively (Table 3). The difference in apparent affinities of vhpA compared to vhpB or C could be due to the stability of the protein and the sequence variation noted among the proteins (Fig. 1A and Table 3). However, the apparent *K_D_* values for the interaction between the Fg-D fragment and the vhp isoforms was around 10-fold higher than binding to full-length Fg, indicating that the Fg D fragment contains a partial binding site for the vhp proteins.

### The Fg-binding motif of vWbp-C encompasses residues 386 to 482.

**FIG 5:**
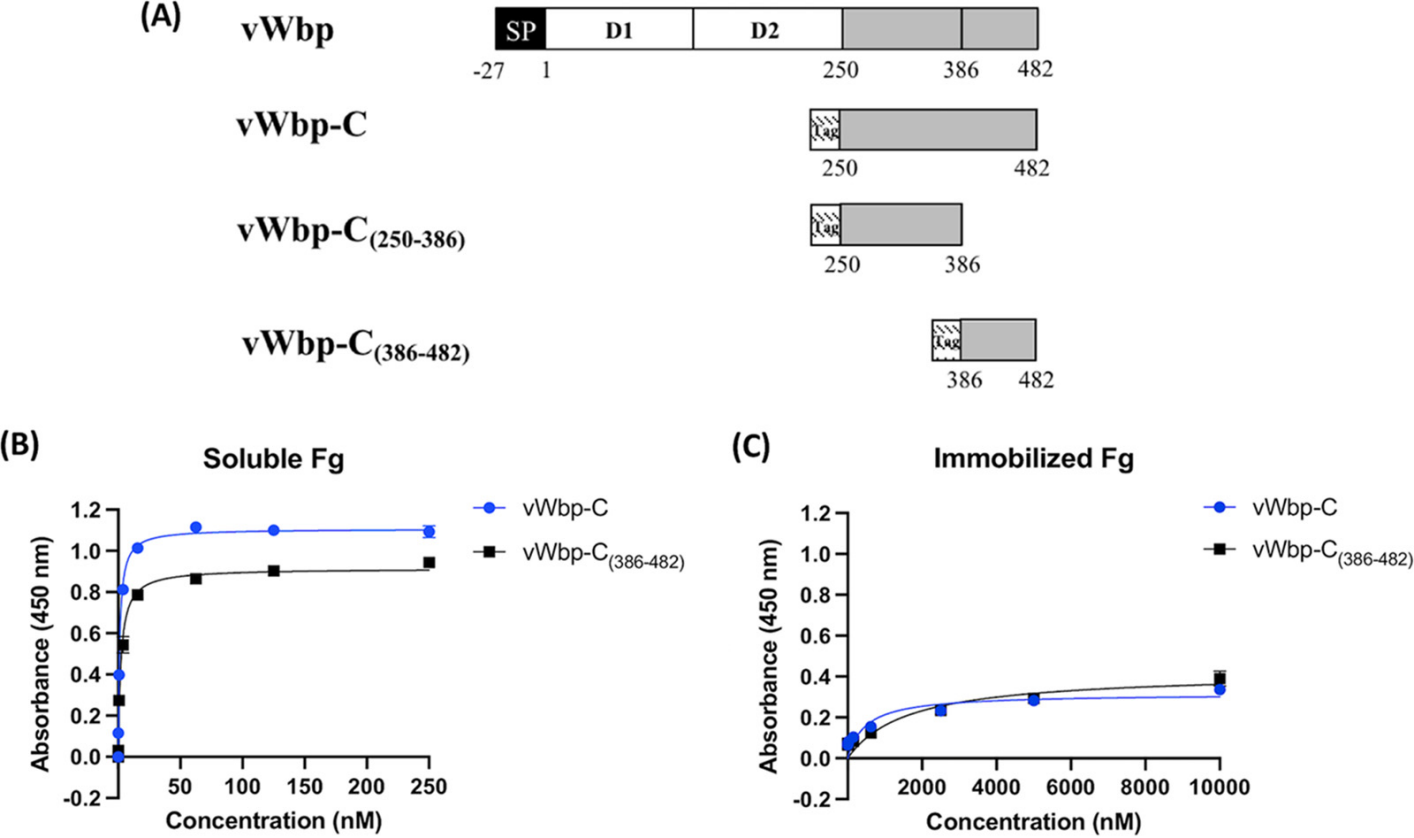
The C-terminal of vWbp harbors an Fg-binding site at residues 386 to 482. (A) Schematic overview of the vWbp constructs generated in this study. Gray represents the predicted unordered region; SP, signal peptide; D_1_D_2_, prothrombin-binding domain; Tag, N-terminal His_6_-MBP tag. (B) ELISA binding of soluble Fg to immobilized vWbp constructs (0.5 μg/well). (C) ELISA binding of vWbp constructs to immobilized Fg (0.5 μg/well).

We observed that amino acid residues ∼386 to 482 of the C-terminal half of vWbp share high sequence identity with the vhp isoforms. Considering that both vWbp-C and vhp bind Fg, we speculated that amino acids 386 to 482 of vWbp might be responsible for Fg binding activity observed in the C-terminal half of the protein. We therefore expressed and purified the C-terminal region of vWbp, covering amino acid region 250 to 482 (vWbp-C) (27), and its two truncated versions, 250 to 386 (vWbp-C_[250 to 386]_) and 386 to 482 (vWbp-C_[386 to 482]_) (Fig. 5A and Fig. S2A). Using the ELISA-type binding assay, we determined that both vWbp-C and vWbp-C_(386 to 482)_ bind soluble Fg in a dose-dependent process that exhibited saturation kinetics (Fig. 5B; apparent *K_D_* of 1.5 nM and 2.4 nM, respectively). Consistent with our earlier published data, vWbp-C showed weak binding to immobilized Fg (Fig. 5C and Table 3) (27). As expected, vWbp-C_(386 to 482)_ also showed little to no binding to immobilized Fg (Fig. 5C). Lastly, vWbp-C_(250 to 386)_ did not bind to either form of Fg under the experimental conditions used (Fig. S2D and E). His_6_-MBP fusion protein was used as a negative control and showed no binding to both soluble and immobilized Fg (Fig. S2D and E). We concluded that the Fg-binding motif for the C-terminal half of vWbp is located at amino acid residues 386 to 482. Furthermore, this motif binds well to soluble Fg but not immobilized Fg, suggesting that vWbp binding to Fg requires a specific structural conformation of Fg that may not be available when Fg is immobilized in the microtiter well.

## DISCUSSION

In this study, we identified a unique protein, vhp, which shows significant sequence identity to the Fg-binding C-terminal section of vWbp (Fig. 1A and B). S. aureus expresses multiple Fg-binding proteins, most of which are classified as MSCRAMMs (3, 17, 18) or SERAMs (3, 6, 17, 19–21). With the exception of vWbp, vhp does not share significant protein sequence identity or a notable motif with other Fg-binding proteins in S. aureus. Analysis of publicly available genomes of S. aureus clinical isolates revealed the presence of three distinct vhp isoforms A, B, and C. We identified Fg as a binding partner for all three isoforms. An initial characterization of this interaction showed that the vhps bind Fg with high affinity. The Fg D fragment, but not the E fragment, bound to the vhps, an observation that locates a vhp interactive site in Fg.

The sequence in the N-terminal half of the vhps shows a high degree of sequence variation between the isoforms. However, within each isoform group, the N-terminal region is conserved across different isolates (Fig. S1A in the supplemental material). This suggests that the N-terminal region of the isoforms might have different functions, perhaps interacting with different targets. The sequences of the C-terminal half of the vhps are conserved among isoforms of isolates and are highly homologous to residues 386 to 482 of vWbp. This vWbp segment was shown to contain a Fg-binding site. Since the prototypes of each vhp isoform behave similarly in their binding to Fg, it seems reasonable to conclude that Fg binding activity is located to the C-terminal region of all vhp isoforms.

Despite the similarities between vWbp and vhp, there are some significant differences in Fg binding between the two staphylococcal proteins. The apparent *K_D_* of vWbp_(386 to 482)_ for binding to soluble Fg is about 10-fold lower than that shown for the binding of the vhp isoforms to Fg (Table 3). The vhp proteins bind the Fg D fragment with an apparent *K*_D_ of 10^−7^ M, whereas vWbp_(386 to 482)_ does not show any significant binding to the Fg D fragment under similar experimental conditions (Table 3) (data not shown). The vhps bind to Fg in the ELISA-type assay when Fg is adsorbed onto the microtiter plate and vhps are added in solution and when the assay is reversed such that the vhps are adsorbed on the plate and Fg is added in solution (Fig. 3). On the other hand, vWbp_(386 to 482)_ only binds to Fg when Fg is in solution. Soluble vWbp_(386 to 482)_ does not show significant binding to Fg adsorbed on the plate (Fig. 5). These observations could indicate that the vhps and vWbp_(386 to 482)_ bind Fg by different mechanisms. An alternative explanation is based on earlier studies by us and others, suggesting that the Fg binding of the C-terminal section of vWbp involves significant conformational arrangements in the two proteins (10, 27, 33, 39). This type of structural rearrangement could be inhibited when a protein is adsorbed in a microtiter plate in which protein flexibility may become restricted. To further define the Fg binding mechanism(s) in vhp and vWbp, the predicted conformational plasticity in the proteins needs to be examined.

vWbp along with Coa and extracellular fibrinogen-binding protein (Efb) appear to form a subfamily of functionally related secreted staphylococcal proteins where the members contribute to host defense evasion (3, 6, 20, 33). These multidomain molecules share homologous domains among each other (40). Both Coa and Efb have similar Fg-binding motifs that map to the intrinsically disordered regions of the proteins (3, 6). Coa and vWbp share a 30% amino acid identity at their N-terminal prothrombin-binding domains (30, 33, 34). They activate prothrombin by a similar mechanism, where the two N-terminal amino acid residues Ile^1^-Val^2^ and Val^1^-Val^2^, respectively, are inserted into the Ile^16^ pocket of prothrombin (33, 34). The now described vhp also fits into this family, as its C-terminal domain is highly homologous to the C-terminal Fg-binding section of vWbp. In addition, the extracellular complement-binding protein (Ecb) is related to Efb and shows 33% sequence identity to the C-terminal C3-binding domain of Efb, covering amino acid residues 65 to 136 of Efb (41, 42). Like *vWb* and *vhp*, the *ecb* and *efb* genes are located close to each other. Both *efb* and *ecb* genes are encoded in a mobile genetic element called IEC-2 (42).

The *vhp* gene is located within a gene cluster that includes genes coding for clumping factor A (ClfA), vWbp, extracellular matrix protein (Emp), and Nuc proteins (Fig. 2). The other proteins encoded in this cluster are identified as virulence factors that act by neutralizing the host defense system (1, 22, 25, 35, 37, 43). The location of *vhp* in the S. aureus genome raises a number of questions about the protein. Since all of the other proteins in this cluster are involved in evading host defenses, does vhp also participate in immune evasion? If yes, how does its interaction with Fg contribute to immune evasion? To what extent does vhp cooperate in its actions with the different proteins in this gene cluster? A possible functional cooperation among these proteins would require coordinated expression. As an example of this cooperation, ClfA and vWbp have been noted to interact with each other, resulting in enhanced adherence of S. aureus to the endothelial vessel wall (44, 45). These and related questions will be topics for future studies.

## MATERIALS AND METHODS

### Bacterial strains and culture conditions.

Escherichia coli strain Rosetta2(DE3) (Novagen) was used as the host for plasmid cloning and expression of His_6_-MBP-tagged recombinant proteins. For the His_6_-MBP-tagged fusion proteins, E. coli was grown on Terrific broth medium (Invitrogen) supplemented with kanamycin (50 μg/ml) and chloramphenicol (34 μg/ml).

### Cloning of vhp and vWbp constructs.

The *vhp* and *vwb* genes from S. aureus strain Newman were codon optimized and synthesized by Twist Biosciences (San Francisco, CA) as gene fragments. N-terminal His_6_-MBP constructs vhpA/B/C/vWbp-C/vWbp-C_(386 to 482)_/or vWbp-C_(250 to 386)_ were generated using synthesized *vhp* or *vWb* gene fragments and were cloned into a pET28b (Agilent Technologies) plasmid. Fragments were digested with BamHI and Xhol using an In-Fusion cloning kit (TaKaRa Bio), resulting in the expression of N-terminal His_6_-MBP constructs under the T7 *lac* promoter. Integrity of the resulting plasmid was confirmed by DNA sequencing.

### Expression and purification of recombinant proteins.

E. coli strains containing plasmids pET28b-vhpA/B/C/vWbp-C/vWbp-C_(386 to 482)_/or vWbp-C_(250 to 386)_ were grown overnight in Terrific broth medium containing chloramphenicol (34 μg/ml) and kanamycin (50 μg/ml) at 37°C. The overnight cultures were used to inoculate fresh Terrific broth medium containing kanamycin (50 μg/ml) and grown to an optical density at 600 nm (OD_600_) of 0.8. After the culture was chilled to 4°C, recombinant expression was induced with 0.5 mM isopropyl 1-thio-β-d-galactopyranoside for 22 h at 22°C. Cells were harvested and resuspended in 50 mM Tris-HCl (pH 8.0) containing 300 mM NaCl, 20 mM imidazole, and 10% glycerol. The homogenous suspensions were centrifuged and lysed with two passes through an M-110p microfluidizer (Microfluidics International Corp.) at 20,000 lb/in^2^ and then centrifuged at 30,000 × *g* for 1 h at 4°C. Supernatants were purified using a HisTrap HP 5-ml column (GE Healthcare), washed with phosphate-buffered saline (PBS; Gibco; pH 7.4) containing 35 mM imidazole, and eluted with 500 mM imidazole. Imidazole was removed by using a HiPrep 26/10 desalting column (GE Healthcare). Peak fractions were then loaded onto an MBPTrap HP 5-ml column (GE Healthcare) and eluted with PBS containing 10 mM maltose. All purifications were conducted on the ÄKTA pure system (GE Healthcare).

### Gel permeation chromatography.

Purified peak fractions from MBPTrap were further purified by loading onto a HiLoad 16/600 Superdex 200 pg column (GE Healthcare) preequilibrated with PBS. The fractions were pooled and concentrated using Amicon Ultra-15 centrifugal filters (10,000 nominal molecular weight limit [NMWL]) (Merck Millipore). Purification was conducted on the ÄKTA pure system. Protein purity was analyzed on SDS-PAGE gels stained with Coomassie blue R-250 (Sigma-Aldrich). Protein concentration was determined by a detergent-compatible protein assay with a Bio-Rad kit (Hercules, CA) using bovine serum albumin (BSA) as the standard.

### Identification of vhp and isoforms.

The vhp proteins were identified using BLAST. The amino acid sequence of vWbp, covering residues 250 to 482, was used to query bacterial genomes in the NCBI database. The presence of a vWbp-like open reading frame (ORF) was previously noted but not further explored (30). Analysis of the results was restricted to strains with finished genomes. Identified proteins were considered for analysis if they harbored a signal sequence, which was predicted by using SignalP-5.0 Server, and lacked both the prothrombin-binding and vWf-binding motifs of vWbp. Location of the *vhp* gene in the genome was determined by using the vhp amino acid sequence to search within the published genome as well as by identifying the proteins surrounding the sequence. A set of 33 S. aureus isolates representing multiple STs and with fully assembled genomes in publicly available databases (NCBI or Pathosystems Resource Integration Center [PATRIC]) were selected for further analysis. The vhp protein sequences were extracted from their respective genomes manually and aligned using Clustal Omega (46).

### Fibrinogen.

Human fibrinogen (FIB3, Enzyme Research Laboratories) was used in all experiments.

### ELISA-type binding assay.

Immulon 96-well microtiter plates (4HBX, Thermo Fisher Scientific) were used. Wells were coated overnight at 4°C with 100 μl of 5 μg/ml Fg/Fg-D/Fg-E or His_6_-MBP-vWbp-C/vWbp-C_(386 to 482)_/vWbp-C_(250 to 386)_/vhpA/B/C (diluted in PBS). Plates were blocked with 3% BSA in Tris-buffered saline (TBS) (25 mM Tris, pH 7.4, 3 mM KCl, and 140 mM NaCl). For soluble Fg binding, diluted Fg (in 1% BSA, 0.05% Tween 20, and TBS) was added to coated wells of either the vWbp-C, vWbp-C_(386 to 482)_, vWbp-C_(250 to 386)_, or vhp proteins. For immobilized Fg binding, diluted recombinant vWbp-C, vWbp-C_(386 to 482)_, vWbp-C_(250 to 386)_, or vhp proteins (in 1% BSA, TBS, and 0.05% Tween 20) were added to the Fg/Fg-D/Fg-E-coated wells and incubated for 1 h at room temperature. Bound Fg was detected using horseradish peroxidase (HRP)-conjugated human Fg polyclonal antibodies (1:1,000 dilution; Rockland Immunochemicals, Inc.). HRP-conjugated anti-His polyclonal antibodies (1:3,000 dilution; R&D Systems) were used to detect bound recombinant proteins. Binding was quantified by the addition of substrate *o*-phenylenediamine dihydrochloride (Sigma-Aldrich), and the absorbance was measured at 450 nm using the ELISA microtiter plate reader (Biotek Cytation 5). A one-site binding equation was used to fit raw data, and apparent *K_D_* values and goodness of fit (*R*^2^) were obtained from GraphPad Prism software version 8.4.3. Apparent *K_D_* values represent averages of three independent experiments.

### Preparation of Fg fragments.

Fg (Enzyme Research Laboratories) was digested with plasmin (10 μg/15 mg of Fg) (Enzyme Research Laboratories) in TBS containing 10 mM CaCl_2_ for 4 h at 37°C as previously described (47) with modifications. Following plasmin digestion, Fg D fragment was obtained by column gel filtration on a Sephacryl S-200 (GE Healthcare) column with a molecular weight cutoff of 30 K, followed then by filtration in a column with a 50-K cutoff to remove plasmin. Purity of purified D fragments was analyzed by SDS-PAGE and appeared as a single band with a molecular mass of 85 kDa. Human Fg E fragment (Fg-E) was purchased from Haematologic Technologies (HCI-0150E).

### Accession numbers.

vWbp from S. aureus Newman (AAK52333.1), vhpA from S. aureus N315 (BAB41978.1), vhpB from S. aureus USA300_ FPR3757 (ABD21761.1), and vhpC from S. aureus TCH60 (ADQ77850.1) are available from GenBank/EMBL under the accession numbers listed.
