# Supplementary material for: vhp Is a Fibrinogen-Binding Protein Related to vWbp in Staphylococcus aureus
Source: mBio. 2021 Aug 3;12(4):e01167-21. doi: 10.1128/mBio.01167-21 (PMC8406236; doi:10.1128/mBio.01167-21)
Supplement: TABLE S1 [file mbio.01167-21-st001.pdf]

Percent of amino acid identity of conserved C-terminal region of vWbp and vhp

Proteins	vWbp (%)	vhpA (%)	vhpB (%)	vhpC (%)
**$vWbp_{(404\text{-}482)}$**	100	58.23	67.09	54.43
**$vhpA_{(63\text{-}147)}$**	58.23	100	70.37	87.06
**$vhpB_{(50\text{-}130)}$**	67.09	70.37	100	70.37
**$vhpC_{(75\text{-}159)}$**	54.43	87.06	70.37	100

[a.] Percent of amino acid identity of $vWbp_{(404\text{-}482)}$- *S. aureus* Newman (AAK52333.1); vhpA- *S. aureus* N315 (BAB41978.1); vhpB- *S. aureus* USA300_ FPR3757 (ABD21761.1); vhpC-*S. aureus* TCH60 (ADQ77850.1).
