# Supplementary material for: vhp Is a Fibrinogen-Binding Protein Related to vWbp in Staphylococcus aureus
Source: mBio. 2021 Aug 3;12(4):e01167-21. doi: 10.1128/mBio.01167-21 (PMC8406236; doi:10.1128/mBio.01167-21)
Supplement: FIG S1 [file mbio.01167-21-sf001.pdf]

## Fig S1A Sequence alignment of Vhp isoforms

```
CAI80435.1      --------------------------------MKRKVLVLTMGVICATQLWHSNHANALV 28
ANI73772.1      --------------------------------MKRKVLVLTMGVICATQLWHSNHANALV 28
AGO29166.1      --------------------------------MKRKVLVLTMGVICATQLWHSNHANALV 28
ADL22693.1      --------------------------------MKRKVLVLTMGVICATQLWHPNHANALV 28
CCG15434.1      --------------------------------MKRKVLVLTMGVLCATQLWHSNHANALV 28
ADI97346.1      --------------------------------MKRKVLVLTMGVICATQLWHSNHANALV 28
BAB94633.1      --------------------------------MKRKVLVLTMGVICATQLWHSNHANALV 28
AGU60934.1      --------------------------------MKRKVLVLTMGVICATQLWHSNHANALV 28
AMV84570.1      --------------------------------MKRKVLVLTMGVICATQLWHSNHANALV 28
BAB41978.1      --------------------------------MKRKVLVLTMGVICATQLWHSNHANALV 28
BAF77693.1      --------------------------------MKRKVLVLTMGVICATQLWHSNHANALV 28
ABR51687.1      --------------------------------MKRKVLVLTMGVICATQLWHSNHANALV 28
ADC36980.1      --------------------------------MKRKVLVLTMGVICATQLWHSNHANALV 28
AQD18902.1      --------------------------------MKRKVLVLTMGVICATQLWHSNHANALV 28
CCJ10585.1      --------------------------------MKRKVLVLTMGVICATQLWHSNHANALV 28
CAG39854.1      --------------------------------MKRKVLVLTMGVLCATQLWQTNNAKALV 28
ADQ77850.1      --------------------------------MKRKVLVLTMGVLCATQLWQTNNAKALV 28
AIO20462.1      --------------------------------MKRKVLVLTMGVLCATQLWQTNNAKALV 28
CRL33498.1      --------------------------------MKRKVLVLTMGVICATQLWHSNHANALV 28
AFH69102.1      MFLKYWYIIFNGEENRVKKSVFKSKQIRELKQLKRKVLVLTMGVLCATQLWQTNNAKALV 60
ABD29944.1      --------------------------------MKRKVLVLTMGVICATQLWHSNHANALV 28
AEW64856.1      --------------------------------MKRKVLVLTMGVICATQLWHSNHANALV 28
ABD21761.1      --------------------------------MKRKVLVLTMGVICATQLWHSNHANALV 28
AAW36414.1      --------------------------------MKRKVLVLTMGVICATQLWHSNHANALV 28
ELP41198.1      --------------------------------MKRKVLVLTMGVICATQLWHSNHANALV 28
EJE56573.1      -----------------------------------------MGVICATQLWHSNHANALV 19
CBI48744.1      --------------------------------MKRKVLVLTMGVICATQLWHSNHANALV 28
AEV77849.1      --------------------------------MKRKVLVLTMGIICATQLWHSNHVNALV 28
ALQ99050.1      --------------------------------MKRKVLVLTMGIICATQLWHSNHVNALV 28
AGU54604.1      --------------------------------MKRKVLVLTMGIICATQLWHSNHVNALV 28
                                                         **::******: *:.:***

CAI80435.1      TESIETNFGVKDSDNKNILQTHTEITTEEKFSIVEKSQLN-------------------- 68
ANI73772.1      TESVETNFVVKDSDNKNILQTHSEITTEEKFSIVEKSQLN-------------------- 68
AGO29166.1      TESVETNFVVKDSDNKNFLQTHTEITTEEKFSIVEKSQLN-------------------- 68
ADL22693.1      TESIETNFVVKDSDNKNILQTHTEITTEEKFSIVEKSQLN-------------------- 68
CCG15434.1      TESVETNFVVKDSGNNNILQTHTEITTEEKFSIVEKSQLN-------------------- 68
ADI97346.1      TESVETNFVVKDSDNENVLQTHTEITTEEKFSIVEKSQLN-------------------- 68
BAB94633.1      SESVETNFVVKDSDNKNILQTHTEITTEEKFSVVEKSQLN-------------------- 68
AGU60934.1      TESVETNFVVKDSDNKNILQTHTEITTEEKFSVVEKSQLN-------------------- 68
AMV84570.1      TESVETNFVVKDSDNKNILQTHTEITTEEKFSVVEKSQLN-------------------- 68
BAB41978.1      TESVETNFVVKDSDNKNILQTHTEITTEEKFSIVEKSQLN-------------------- 68
BAF77693.1      TESVETNFVVKDSDNKNILQTHTEITTEEKFSIVEKSQLN-------------------- 68
ABR51687.1      TESVETNFVVKDSDNKNILQTHTEITTEEKFSIVEKSQLN-------------------- 68
ADC36980.1      TESVETNFVVKDSDNKNILQTHTEITTEEKFSIVEKSQLN-------------------- 68
AQD18902.1      TESVETNFVVKDSDNKNILQTHTEITTEEKFSIVEKSQLN-------------------- 68
CCJ10585.1      TESVETNFVVKDSDNKNILQTHTEITTQEKFSIVEKSQLN-------------------- 68
CAG39854.1      TESGVND-----------TKQFTEVTSEEKVIKDAISKVNESFIYYPQNDLKGLGGEHND 77
ADQ77850.1      TESGVND-----------TKQFTEVTSEEKVIKDAISKVNESFIYYPQNDLKGLGGEHND 77
AIO20462.1      TESGVND-----------TKQFTEVTSEEKVIKDAISKVNESFIYYPQNDLKGLGGEHND 77
CRL33498.1      TESGAYD-----------TKQFTEIVSEEKVIKDSISKVNESFIYYPQNDLKGLGGEHND 77
AFH69102.1      HESGVND-----------TKQFTEVTSEEKVITVEHAYIN-------------------- 89
ABD29944.1      TESGAND-----------TKQFTEIVSEEKVITVEHAQIN-------------------- 57
AEW64856.1      TESGAND-----------TKQFTEIVSEEKVITVEHAQIN-------------------- 57
ABD21761.1      TESGAND-----------TKQFTEIVSEEKVITVEHAQIN-------------------- 57
AAW36414.1      TESGAND-----------TKQFTEIVSEEKVITVEHAQIN-------------------- 57
ELP41198.1      TESGAND-----------TKQFTEIVSEEKVITVEHAQIN-------------------- 57
EJE56573.1      TESGAND-----------TKQFTEIVSEEKVITVEHAQIN-------------------- 48
CBI48744.1      TESGAND-----------TKQFTEIVSEEKVITVEHAQIN-------------------- 57
```

```
AEV77849.1      TESGAND-----------TKQFTEIVSEEKVITVEHAQIN--------------------- 57
ALQ99050.1      TESGAND-----------TKQFTEIVSEEKVITVEHAQIN--------------------- 57
AGU54604.1      TESGAND-----------TKQFTEIVSEEKVITVEHAQIN--------------------- 57
                 **   :            : .:*:.::**.     : :*

CAI80435.1      ---KLKSLSNDNYIEYDLHTNQTGIKKGWLYGYSEIDSSHFTDRDKRVIRRDHVKEAQSL 125
ANI73772.1      ---KLKALSNDNYIEYDLHTNQTGIKKGWLYGYSEIDSSHFTDRDKRVIRRDHVKEAQSL 125
AGO29166.1      ---TLKSLSNDNYIEYDLHTNQTGSKKGWLYGYSEIDSSHFTDRDKRAIRRDHVKEAQNL 125
ADL22693.1      ---KLKSLSNDNYIEYDLHTNQTGIKKGWLYGYSEIDSSHFTDRDKRVIRRDHVKEAQSL 125
CCG15434.1      ---TLKSLSNDNYIEYDLHTNQTGIKKGWLYGYSEIDSSHFTDCDKRAIRRDHVKEAQSL 125
ADI97346.1      ---KLKSLSNDNYIEYDLHTNQTGIKKGWLYGYSEIDSSHFTDRDKRVIRRDHVKEAQSL 125
BAB94633.1      ---TLKSLSNDNYIEYDLHTNQTGIKKGWLYGYSEIDSSHFTDRDKRVIRRDHVKEAQSL 125
AGU60934.1      ---TLKSLSNDNYIEYDLHTNQTGIKKGWLYGYSEIDSSHFTDRDKRVIRRDHVKEAQSL 125
AMV84570.1      ---TLKSLSNDNYIEYDLHTNQTGIKKGWLYGYSEIDSSHFTDRDKRVIRRDHVKEAQSL 125
BAB41978.1      ---KLKALSNDNYIEYDLHTNQTGIKKGWLYGYSEIDSSHFTDRDKRVIRRDHVKEAQSL 125
BAF77693.1      ---KLKALSNDNYIEYDLHTNQTGIKKGWLYGYSEIDSSHFTDRDKRVIRRDHVKEAQSL 125
ABR51687.1      ---KLKALSNDNYIEYDLHTNQTGIKKGWLYGYSEIDSSHFTDRDKRVIRRDHVKEAQSL 125
ADC36980.1      ---KLKALSNDNYIEYDLHTNQTGIKKGWLYGYSEIDSSHFTDRDKRVIRRDHVKEAQSL 125
AQD18902.1      ---KLKALSNDNYIEYDLHTNQTGIKKGWLYGYSEIDSSHFTDRDKRVIRRDHVKEAQSL 125
CCJ10585.1      ---KLKALSNDNYIEYDLHTNQTGIKKGWLYGYSEIGSSHFTDRDKRVIRRDHVKEAQSL 125
CAG39854.1      YEKITYSTSSNNVLELSMSSKYVGGKSGAMVGYSEIYSSHFTDRDKRAIRRDHVKEAQNL 137
ADQ77850.1      YEKITYSTSSNNVLELSMSSKYVGGKSGAMVGYSEIYSSHFTDRDKRAIRRDHVKEAQNL 137
AIO20462.1      YEKITYSTSSNNVLELSMSSKYVGGKSGAMVGYSEIYSSHFTDRDKRAIRRDHVKEAQNL 137
CRL33498.1      YEKITYSTSSNSVLEISMSSNYVGGKSGAMVGYSEIDSSHFTDRDKRVIRRDHVKEAQNL 137
AFH69102.1      ---IFKSNSNSNLME--FNILTMGGKSGAMVGYSEIDSSHFTDRDKRAIRRDHVKEAQSL 144
ABD29944.1      ---IFQSNSNSNLME--FNILTMGGKSGAMVGYSEIDSSHFTDRDKRVIRRDHVKEAQSL 112
AEW64856.1      ---IFQSNSNSNLME--FNILTMGGKSGAMVGYSEIDSSHFTDRDKRVIRRDHVKEAQSL 112
ABD21761.1      ---IFQSNSNSNLME--FNILTMGGKSGAMVGYSEIDSSHFTDRDKRVIRRDHVKEAQSL 112
AAW36414.1      ---IFQSNSNSNLME--FNILTMGGKSGAMVGYSEIDSSHFTDRDKRVIRRDHVKEAQSL 112
ELP41198.1      ---IFQSNSNSNLME--FNILTMGGKSGAMVGYSEIDSSHFTDRDKRVIRRDHVKEAQSL 112
EJE56573.1      ---IFQSNSNSNLME--FNILTMGGKSGAMVGYSEIDSSHFTDRDKRVIRRDHVKEAQSL 103
CBI48744.1      ---IFQSNSNSNLME--FNILTMGGKSGAMVGYSEIDSSHFTDRDKRVIRRDHVKEAQSL 112
AEV77849.1      ---NFKSNSNSNLME--FNILTMGGKSGAMVGYSEIDSSHFTDRDKRVIRRDHVKEAQSL 112
ALQ99050.1      ---NFKSNSNSNLME--FNILTMGGKSGAMVGYSEIDSSHFTDRDKRVIRRDHVKEAQSL 112
AGU54604.1      ---NFKSNSNSNLME--FNILTMGGKSGAMVGYSEIDSSHFTDRDKRVIRRDHVKEAQSS 112
                      : *... :*  :     * *.* : ***** ****** ***.**********.

CAI80435.1      VENYKDTQSADARMKAKQKVNTLSKPHQNYFNKQIDKVYNGLQR----    169
ANI73772.1      VENYKDIQSAEARMKAKQKVNTLSKPHQNYFNKQIDKVYNGLQR----    169
AGO29166.1      INDYKYTQKYEDFAKATAKVSTLSLSHQNYLNKQIDKVNNKIEKTEKP    173
ADL22693.1      INDYKYTQKYEDFAKATAKVSTLSQSHQNYLNKQIDNVNNQIEKTEKR    173
CCG15434.1      INDYKDTQSYEDLAKATAKVSTLSQSHQNYLNKQIDKVNNKIEKTEKR    173
ADI97346.1      INDYKDTQSYEDLAKATAKVSTLSQSHQNYLNKQIDKVNNKIEKTEKR    173
BAB94633.1      INDYKYTQKYEDLAKATAKVSTLSQSHQNYLNKQIDKVNNKIEKTEKR    173
AGU60934.1      INDYKDTQSYEDLAKATAKVSTLSQSHQNYLNKQIDKVNNKIEKTEKR    173
AMV84570.1      INDYKDTQSYEDLAKATAKVSTLSQSHQNYLNKQIDKVNNKIEKTEKR    173
BAB41978.1      INDYKDTQSYEDLAKATAKVSTLSQSHQNYLNKQIDKVNNKIEKTEKR    173
BAF77693.1      INDYKDTQSYEDLAKATAKVSTLSQSHQNYLNKQIDKVNNKIEKTEKR    173
ABR51687.1      INDYKDTQSYEDLAKATAKVSTLSQSHQNYLNKQIDKVNNKIEKTEKR    173
ADC36980.1      INDYKDTQSYEDLAKATAKVSTLSQSHQNYLNKQIDKVNNKIEKTEKR    173
AQD18902.1      INDYKDTQSYEDLAKATAKVSTLSQSHQNYLNKQIDKVNNKIEKTEKR    173
CCJ10585.1      INDYKDTQSYEDLAKATAKVSTLSQSHQNYLNKQIDKVNNKIEKTEKR    173
CAG39854.1      INDYKYTQIYEDFAKATAKVSTLSQSHQNYLNKQIDKVNNKIEKTEKR    185
ADQ77850.1      INDYKYTQIYEDFAKATAKVSTLSQSHQNYLNKQIDKVNNKIEKTEKR    185
AIO20462.1      INDYKYTQIYEDFAKATAKVSTLSQSHQNYLNKQIDKVNNKIEKTEKR    185
CRL33498.1      INDYKYTQKYEDLAKATAKVSTLSQSHQNYLNKQIDKVNNKIEKTEKR    185
AFH69102.1      INDYKDTQSYEDLAKATAKVSTLSQSHQNYLNKQIDKVNNKIEKTEKR    192
ABD29944.1      VENYKDTQSADARMKAKQKVNTLSKPHQNYFNKQIDKVYNGLQR----    156
AEW64856.1      VENYKDTQSADARMKAKQKVNTLSKPHQNYFNKQIDKVYNGLQR----    156
ABD21761.1      VENYKDTQSADARMKAKQKVNTLSKPHQNYFNKQIDKVYNGLQR----    156
AAW36414.1      VENYKDTQSADARMKAKQKVNTLSKPHQNYFNKQIDKVYNGLQR----    156
ELP41198.1      VENYKDTQSADARMKAKQKVNTLSKPHQNYFNKQIDKVYNGLQR----    156
EJE56573.1      VENYKDTQSADARMKAKQKVNTLSKPHQNYFNKQIDKVYNGLQR----    147
```

| | | |
|---|---|---|
| CBI48744.1 | VENYKDTQSADARMKAKQKVNTLSKPHQNYFNKQIDKVYNGLQR---- | 156 |
| AEV77849.1 | VENYKDTQSADARMKAKQKVNTLSKPHQNYFNKQIDKVYNGLQR---- | 156 |
| ALQ99050.1 | VENYKDTQSADARMKAKQKVNTLSKPHQNYFNKQIDKVYNGLQR---- | 156 |
| AGU54604.1 | ------------------------------------------------ | 112 |

## Fig S1B. Sequence alignment of vWbp isoforms

```
AMV79382.1      MKNKLLVLSLGALCVSQIWESNRASAVVSGEKNPYVSKALSVSGQKSNNWSLEQYKESLN 60
AFH69100.1      MKNKLLVLSLGALCVSQIWESNRASAVVSGEKNPYVSESLKLNDNKSKSLSLEKYKESLR 60
AMV84568.1      MKNKLLVLSLGALCVSQIWESNHASAVVSGEKNPYVSESLKLNDNKSKSLSLEKYKESLR 60
CRL33493.1      MKNKLLVLSLGALCVSQIWESNRASAVVSGEENPYVSESLKLNDNKSKSLSLEKYKESLR 60
ANI73770.1      MKNKLLVLSLGALCVSQIWESNRASAVVSGEENPYVSKALSVSGQKSNNLTLKQYKDSLR 60
EJE56571.1      MKNKLLVLSLGALCVSQIWESNHASAVVSGEKNPYVSKALSIKGQKTNSWNLGQYKDSLR 60
CAI80433.1      MKNKLLVLSLGALCVSQIWESNRASAVVSGEENPYVSKALSIKGQKTNSWNLGQYKDSLR 60
CCG15432.1      MKNKLLVLSLGALCVSQIWESNHASAVVSGEKNPYVSKALSIKGQKTNSWNLGQYKDSLR 60
AEV77847.2      MKNKLLVLSLGALCVSQIWESNRASAVVSGEENPYVSKALSVSGQKSNNLTLKQYKDSLR 60
ALQ99048.1      MKNKLLVLSLGALCVSQIWESNRASAVVSGEENPYVSKALSVSGQKSNNLTLKQYKDSLR 60
BAF77691.1      MKNKLLVLSLGALCVSQIWESNHASAVVSGEKNPYVSKALELKDKSNKSNSYENYRDSLE 60
AQD18904.1      MKNKLLVLSLGALCVSQIWESNHASAVVSGEKNPYVSKALELKDKSNKSNSYENYRDSLE 60
ABR51685.1      MKNKLLVLSLGALCVSQIWESNHASAVVSGEKNPYVSKALELKDKSNKSNSYENYRDSLE 60
ADC36978.1      MKNKLLVLSLGALCVSQIWESNHASAVVSGEKNPYVSKALELKDKSNKSNSYENYRDSLE 60
CCJ10583.1      MKNKLLVLSLGALCVSQIWESNHASAVVSGEKNPYVSKALELKDKSNKSNSYENYRDSLE 60
ADI97344.1      MKNKLLVLSLGALCVSQIWESNRASAVVSGEKNPYVSKALELKGTSNKSNTYENYRESLE 60
ABD20992.1      MKNKLLVLSLGALCVSQIWESNRASAVVSGEKNPYVSESLKLTNNKNKSRTVEEYKKSLD 60
CBI48742.1      MKNKLLVLSLGALCVSQIWESNRASAVVSGEKNPYVSESLKLTNNKNKSRTVEEYKKSLD 60
AAW36412.1      MKNKLLVLSLGALCVSQIWESNRASAVVSGEKNPYVSESLKLTNNKNKSRTVEEYKKSLD 60
ELP41199.1      MKNKLLVLSLGALCVSQIWESNRASAVVSGEKNPYVSESLKLTNNKNKSRTVEEYKKSLD 60
AGO29164.1      MKNKLLVLSLGALCVSQIWESNRASAVVSGEKNPYVSESLKLTGSKSTGVTPAMYKENLE 60
ADQ77852.1      MKNKLLVLSLGALCVSQIWESNRASAVVSGEENPYKSESLKLNGKRSTTITSDKYEENLD 60
AIO20460.1      MKNKLLVLSLGALCVSQIWESNRASAVVSGEENPYKSESLKLNGKRSTTITSDKYEENLD 60
AEW64854.1      MKNKLLVLSLGALCVSQIWESNRASAVVSGEENPYKSESLKLNGKRSTTITSDKYEENLD 60
AGU54602.1      MKNKLLVLSLGALCVSQIWEYNHASAVVSGEKNPYKSESLKLTGKRSNSVTPVMYKENLE 60
ADL22691.1      MKNKLLVLSLGALCVSQIWESNRASAVVSGEKNPYVSESLKLTGSKSTVVTPAMYKENLE 60
                ******************** *:********:*** *::*.:..  ..  .   *...*

AMV79382.1      KLITSIDIQDHDGYDELEYKDINEKYQKKFLAEIDALNKFIDEERKIAYYSKN--NIQIP 118
AFH69100.1      SIMCTKEINKNDGYDEPEYKEALDTYRKKLFAELDALNKFLDEERKIASYIKK--NMEVP 118
AMV84568.1      SIMCTKEINKNDGYDEPEYKEALNTYRKKLFAELDALNKFLDEERKIASYIKK--NMEVP 118
CRL33493.1      SIMCTSEINKNDGYDEPEYKEAMDTYRKKLFAELDALNKFLDEERKIASYIKK--NMEVP 118
ANI73770.1      SVMCTSEINKNDGYDEPEYKEAMDTYRKKLFAELDALNKFLDEERTITIKKKS--NENVS 118
EJE56571.1      TVMCTSEINKNDGYDEPEYKEAMDTYRKKLFAELDALNKFLDEERKIESSKKN--NNQVA 118
CAI80433.1      TVMCTSEINKNDGYDEPEYKEAMDTYRKKLFAELDALNKFLDEERKIESSKKN--NNQVP 118
CCG15432.1      TVMCTSEINKNDGYDEPEYKEAMDTYRKKLFAELDALNKFLDEERKIESSKKN--NNQVA 118
AEV77847.2      SVMCTSEINKNDGYDEPEYKEAMDTYRKKLFAELDALNKFLDEERKIESSKKN--NNQVP 118
ALQ99048.1      SVMCTSEINKNDGYDEPEYKEAMDTYRKKLFAELDALNKFLDEERKIESSKKN--NNQVP 118
BAF77691.1      SLISSLSFADYEKYEEPEYEKAVKKYQQKFMAEDDALKNFLNEEKKIKNADI----SRKS 116
AQD18904.1      SLISSLSFADYEKYEEPEYEKAVKKYQQKFMAEDDALKNFLNEEKKIKNADI----SRKS 116
ABR51685.1      SLISSLSFADYEKYEEPEYEKAVKKYQQKFMAEDDALKNFLNEEKKIKNADI----SRKS 116
ADC36978.1      SLISSLSFADYEKYEEPEYEKAVKKYQQKFMAEDDALKNFLNEEKKIKNADI----SRKS 116
CCJ10583.1      SLISSLSFADYEKYEEPEYEKAVKKYQQKFMAEDDALKNFLNEEKKIKNADI----SRKS 116
ADI97344.1      NLIFSLSFADYEKYEEPEYNNAVKKYQQKFMAEDDALKTFLSEEKKLEKTDS----SRNS 116
ABD20992.1      DLIWSFPNLDNERFDNPEYKEAMKKYQQRFMAEDEALKKFFSEEKKIKNGN------TDN 114
CBI48742.1      DLIWSFPNLDNERFDNPEYKEAMKKYQQRFMAEDEALKKFFSEEKKIKNGN------TDN 114
AAW36412.1      DLIWSFPNLDNERFDNPEYKEAMKKYQQRFMAEDEALKKFFSEEKKIKNGN------TDN 114
ELP41199.1      DLIWSFPNLDNERFDNPEYKEAMKKYQQRFMAEDEALKKFFSEEKKIKNGN------TDN 114
AGO29164.1      SLISSLSFADYEKYEEPEYKEAVKKYQQKFMAEDDALKKFFSEEKKIKNRNTNT----NT 116
ADQ77852.1      MLISSLSFADYEKYEEPEYKEAVKKYQQKFMAEDDALKKFFSEEKKIKNRNT------NT 114
AIO20460.1      MLISSLSFADYEKYEEPEYKEAVKKYQQKFMAEDDALKKFFSEEKKIKNRNT------NT 114
AEW64854.1      MLISSLSFADYEKYEEPEYKEAVKKYQQKFMAEDDALKKFFSEEKKIKNRNTNTNTNTNT 120
AGU54602.1      KLIASLSFADYEKYYEPEYKEAVKTYQQKFMAEDAALKKFFREEKHIKE-------NTNT 113
ADL22691.1      SLISSLSFADYEKYYEPEFKDVVKKYQQRFMAEDAALKKFFREEYFIKE-------NKNS 113
                 :: :    . : : : *::.  ..*:::::**  **:.*: **  :

AMV79382.1      ADILGLTHERYLKIYESLQENKKEFDIESKKIEEKHPELKRYGNEEEYQANVKLNELENK 178
AFH69100.1      SNILGLTHGRYTAIYNAIKENKKEFEKNVENIESKHLDLKRFDEDKDYEARVKLNELENK 178
AMV84568.1      SNILGLTHGRYTAIYNAIKENKNEFEKNVEKIESKHLDLKRFDEDKDYEARVKLNELENK 178
CRL33493.1      SNILGLTHGRYTAIYNAIKENKKEFEKNVENIESKHLDLKRFDEDKDYEARVKLNELENK 178
ANI73770.1      EDILGLTHQRYAAIHQGIKDNKAEFEKKVESIENKYSDLKKFDEVKDDKVRDELNELENK 178
```

```
EJE56571.1      NGVLGLTHQRYLAIHEAIKENKNEFERKTQDIESRNPDLKKFDRDKDYEVRVKLNELENK 178
CAI80433.1      NVVLGLTHQRYLAIHEAIKENKNEFERKTQDIESRNPDLKKFDRDKDYEVRVKLNELENK 178
CCG15432.1      NGVLGLTHQRYLAIHEAIKENKNEFERKTQDIESRNPDLKKFDRDKDYEVRVKLNELENK 178
AEV77847.2      NGVLGLTHQRYLAIHEAIKENKNEFERKTQDIESRNPDLKKFDRDKDYEVRVKLNELENK 178
ALQ99048.1      NGVLGLTHQRYLAIHEAIKENKNEFERKTQDIESRNPDLKKFDRDKDYEVRVKLNELENK 178
BAF77691.1      NNLLGLTHERYSYIFDTLKKNKQEFLKDIEEIQLKNSDLKDFNNTEQHNADVEINNLENK 176
AQD18904.1      NDLLGLTHERYSYIFDTLKKNKQEFLKDIEEIQLKNSDLKDFNNTEQHNADVEINNLENK 176
ABR51685.1      NDLLGLTHERYSYIFDTLKKNKQEFLKDIEEIQLKNSDLKDFNNTEQHNADVEINNLENK 176
ADC36978.1      NDLLGLTHERYSYIFDTLKKNKQEFLKDIEEIQLKNSDLKDFNNTEQHNADVEINNLENK 176
CCJ10583.1      NDLLGLTHERYSYIFDTLKKNKQEFLKDIEEIQLKNSDLKDFNNTEQHNADVEINNLENK 176
ADI97344.1      NGLLGLTHERYTYIFDTLKKNKQEFLQEIQEINLKNSDLKDFNDTEQYNADVEINNLENK 176
ABD20992.1      LDYLGLSHERYESVFNTLKKQSEEFLKEIEDIKKDNPELKDFNEEEQLKCDLELNKLENQ 174
CBI48742.1      LDYLGLSHERYESVFNTLKKQSEEFLKEIEDIKKDNPELKDFNEEEQLKCDLELNKLENQ 174
AAW36412.1      LDYLGLSHERYESVFNTLKKQSEEFLKEIEDIKKDNPELKDFNEEEQLKCDLELNKLENQ 174
ELP41199.1      LDYLGLSHERYESVFNTLKKQSEEFLKEIEDIKKDNPELKDFNEEEQLKCDLELNKLENQ 174
AGO29164.1      SNYLGLTHERYESIYNSLKNHREEFSKEIEEINNKNPELKEYNNEEQTKADTELNTLENQ 176
ADQ77852.1      SNYLGLTHERYESIYNSLKNHREEFSKEIEEINNKNPVLKEYNNEEQTKADTELNTLENQ 174
AIO20460.1      SNYLGLTHERYESIYNSLKNHREEFSKEIEEINNKNPVLKEYNNEEQTKADTELNTLENQ 174
AEW64854.1      SNYLGLTHERYESIYNSLKKHREEFSKEIEEINNKNPELKEYNNEEQTKADTELNTLENQ 180
AGU54602.1      NEMLGLTEERYQHIYDKLKSNNEWFMKEIKNIQERYQDLKDFDEKQQHDADVKINELENK 173
ADL22691.1      DEMLGITQERYKYIYDKLKSNNDEFMREIKEIKDSHPDLKDFDANQQHESDVKINDLENK 173
                   **::. **  :.: ::.:   *  . :.*:     ** :.  :: .   ::* ***:

AMV79382.1      VLMLGYAFFGNK-DARENLYNKLDMIVGLSKNEREDKIPKNKRMFEDRIKDLESIIDEFF 237
AFH69100.1      VLMLGQAFPDKV-DARESLYNKLDMIVGLDNDEIEERHPQNERLLKERVEDLETIIDEFF 237
AMV84568.1      VLMLGQAFPDKV-DARESLYNKLDMIVGLSNDEIEERHPQNERLLKERVEDLETIIDEFF 237
CRL33493.1      VLMLGQAFPDKV-DARESLYNKLDMIVGLSNDEIEERHPQNERLLKERVEDLETIIDEFF 237
ANI73770.1      VLMLGQAFPDKV-EARMDLYNKLDMIVGYSEDEREERHPQNERLYKERVEDLETIIDEFF 237
EJE56571.1      VLMLGHAFPNKH-EARENLYNKLDLIVGRSDDEREERHPQNDRLSKERVEDLESIIDEFF 237
CAI80433.1      VLMLGHAFPNQH-EARENLYNKLDLIVGRSDDEREERHPQNDRLSKERVEDLESIIDEFF 237
CCG15432.1      VLMLGHAFPNKH-EARENLYNKLDLIVGRSDDEREERHPQNDRLSKERVEDLESIIDEFF 237
AEV77847.2      VLMLGHAFPNKH-EARENLYNKLDLIVGRSDDEREERHPQNDRLSKERVEDLESIIDEFF 237
ALQ99048.1      VLMLGHAFPNKH-EARENLYNKLDLIVGRSDDEREERHPQNDRLSKERVEDLESIIDEFF 237
BAF77691.1      VLMVGYTFYNTNKDEVEELYSELDLIVGEVQDKSDKKRAVNQRMLNRKKEDLEFIIDKFF 236
AQD18904.1      VLMVGYTFYNTNKDEVEELYSELDLIVGEVQDKSDKKRAVNQRMLNRKKEDLEFIIDKFF 236
ABR51685.1      VLMVGYTFYNTNKDEVEELYSELDLIVGEVQDKSDKKRAVNQRMLNRKKEDLEFIIDKFF 236
ADC36978.1      VLMVGYTFYNTNKDEVEELYSELDLIVGEVQDKSDKKRAVNQRMLNRKKEDLEFIIDKFF 236
CCJ10583.1      VLMVGYTFYNTNKDEVEELYSELDLIVGEVQDKSDKKRAVNQRMLNRKKEDLEFIIDKFF 236
ADI97344.1      VLMLGYTFFSTYKDEVEELYSELDLIVGEVQDKSDKKRAVNQRMLSRKKEDLESIIDKFF 236
ABD20992.1      ILMLGKTFYQNYRDDVESLYSKLDLIMGYKDEERANKKAVNKRMLENKKEDLETIIDEFF 234
CBI48742.1      ILMLGKTFYQNYRDDVESLYSKLDLIMGYKDEERANKKAVNKRMLENKKEDLETIIDEFF 234
AAW36412.1      ILMLGKTFYQNYRDDVESLYSKLDLIMGYKDEERANKKAVNKRMLENKKEDLETIIDEFF 234
ELP41199.1      ILMLGKTFYQNYRDDVESLYSKLDLIMGYKDEERANKKAVNKRMLENKKEDLETIIDEFF 234
AGO29164.1      VLMIGYTFYHSNKNEVEDLYNKLDMILGYKDEERKKKRATNQRMFNNKKEDLETIIDEFF 236
ADQ77852.1      VLMIGYTFYHSNKNEVEDLYNKLDMILGYKDEERKKKRATNQRMFNNKKEDLETIIDEFF 234
AIO20460.1      VLMIGYTFYHSNKNEVEDLYNKLDMILGYKDEERKKKRATNQRMFNNKKEDLETIIDEFF 234
AEW64854.1      VLMIGYTFYHSNKNEVEDLYNKLDMILGYKDEERKKKRATNQRMFNNKKEDLETIIDEFF 240
AGU54602.1      VLMLGYTFGNVGG-ARTNLYSKLDLILGYEDYERKYKQPTNSRMLNEKMEDLETIIDEFF 232
ADL22691.1      VLMLGYAFKNESE-ARLNLYSKLDLILGYKSYERLYKKPTNSRMLKEKIEDLESIIDEFF 232
                :**:* :*         .**.:**:*:*  . :   :   *.*: . : :*** ***:**

AMV79382.1      VEINENRPLNIPALVESNE--ENIVMAKKLKADTEEAKANT---SKRSKRSLNTQNHKSK 292
AFH69100.1      NDIGENRPENIAPLTNNEH--KNKEMITKLKSDTEAAKSDESKRSKRSKRSLNTQNHKSK 295
AMV84568.1      NDIGENRPENISPLTNNEY--KNKEMITKLKSDTEAAKNDESKRSKRSKRSLNTQNYKSA 295
CRL33493.1      NDIGENRPENISPLTNNEH--KNKEMITKLKADTEEAKTNTA---KRSKRSLNTHNHKST 292
ANI73770.1      KDINENRPANIPALTSDKE--NNRSMALKLKQDTEAAKNDESKRSKRSKRSLNTQNYKSA 295
EJE56571.1      VEINENRPLNIPALVESNE--ENIEMAKKLKADTEEAKTNN---AKRSKRSLNTQNYKSA 292
CAI80433.1      VEINENRPLNIPALVESNE--ENIEMAKKLKADTEEAKTNT---AKRSKRSLNTQNYKSA 292
CCG15432.1      VEINENRPLNIPALIESNE--ENIVMAKKLKADTEEAKANT---SKRSKRSLNTQNHKSK 292
AEV77847.2      VEINENRPLNIPALVESNE--ENIVMAKKLKADTEEAKANT---SKRSKRSLNTQNHKSK 292
ALQ99048.1      VEINENRPLNIPALVESNE--ENIVMAKKLKADTEEAKANT---SKRSKRSLNTQNHKSK 292
BAF77691.1      KKIQQERPESIPALTSEKN--HNQTMALKLKADTEAAKNDVSK---RSKRSLNTQNNKST 291
AQD18904.1      KKIQQERPESIPALTSEKN--HNQTMALKLKADTEAAKNDVSK---RSKRSLNTQNNKST 291
```

```
ABR51685.1      KKIQQERPESIPALTSEKN--HNQTMALKLKADTEAAKNDVSK---RSKRSLNTQNNKST 291
ADC36978.1      KKIQQERPESIPALTSEKN--HNQTMALKLKADTEAAKNDVSK---RSKRSLNTQNNKST 291
CCJ10583.1      KKIQQERPESIPALTSEKN--HNQTMALKLKADTEAAKNDVSK---RSKRSLNTQNNKST 291
ADI97344.1      KEIKQERPENIPALTSDKN--HNQSMALKLKSDTEAAKTDVSK---RSKRSLESQKYKST 291
ABD20992.1      SDIDKTRPNNIPVLEDEKQEEKNHKNMAQLKSDTEAAKSDESKRSKRSKRSLNTQNHKPA 294
CBI48742.1      SDIDKTRPNNIPVLEDEKQEEKNHKNMAQLKSDTEAAKSDESKRSKRSKRSLNTQNHKPA 294
AAW36412.1      SDIDKTRPNNIPVLEDEKQEEKNHKNMAQLKSDTEAAKSDESKRSKRSKRSLNTQNHKPA 294
ELP41199.1      SDIDKTRPNNIPVLEDEKQEEKNHKNMAQLKSDTEAAKSDESKRSKRSKRSLNTQNHKPA 294
AGO29164.1      GEIGQQRPTSIPTLAPKEEKETNTNNANKLKSDTEAAKNDETKRSKRSKRSLNTHNHKSA 296
ADQ77852.1      GEIGQQRPTSIPTLAPKEEKETNIKNANKLKSDTEAAKNDEAK------RSLNTHNHKSV 288
AIO20460.1      GEIGQQRPTSIPTLAPKEEKETNIKNANKLKSDTEAAKNDEAK------RSLNTHNHKSV 288
AEW64854.1      GEIGQQRPTSIPTLAPKEEKETNTNNANKLKYDTEAAKTDESKRSNRSKRSLDSQNYKSV 300
AGU54602.1      EEIGKQRPINIPTLASEKEKETNAKNANKLRADTEDAINDENKRCNRSKRSLNTQNHKPA 292
ADL22691.1      EEINKERPKDIPVLSEYEH--ENKEKAITLKSDTEAAKNDETKRSKRSKRSLNTQNHKSA 290
                 .* : ** .*  *   :    *      *: *** *  :         ***:::: *

AMV79382.1      INEVTEEQKANYEKKFKEIKERFLAKQKYKNNTPVVSLEYDEDDN-----ENDKKLVVSA 347
AFH69100.1      INEVTAEQKAEYERKAEERKEKFLAKNK---DNPVVSLIDDEDDN---ENENDKQLVVSA 349
AMV84568.1      SQEVTAEQKAEYERKAKERKDKFLAKNK---DNPVVSLIDDEDDN-----ENDKKLVVSA 347
CRL33493.1      SREVTAEQKAEYERKAEERKEKFLAKNK---DNPVVSLIDDEDDN-----ENDKQLVISA 344
ANI73770.1      SQEVTAEQKAEYERKAEERKEKFLAKNK---DNPVVSLIDDEDDN-----ENDKQLVVSA 347
EJE56571.1      SQEVTAEQKAEYERKAEERKEKFLAKNK---DNPVVSLIDDEDDN-----ENDKQLVVSA 344
CAI80433.1      SQEVSEQQKAEYERKAEERKEKFLAKNK---DNPVVSLIDDEDDN-----ENDKQLVVSA 344
CCG15432.1      INEVTEEQKANYEKKFKEIKERFLAKQKNKNNTPVVSLEYDEDDN-----ENDKKLVVSE 347
AEV77847.2      INEVTEEQKANYEKKFKEIKERFLAKQKNKNNTPVVSLEYDEDDN-----ENDKQLVVSA 347
ALQ99048.1      INEVTEEQKANYEKKFKEIKERFLAKQKNKNNTPVVSLEYDEDDN-----ENDKQLVVSA 347
BAF77691.1      TQEISEEQKAEYQRKSEALKERFINRQKSK-NESVVSLIDDED-----DNENDRQLVVSA 345
AQD18904.1      TQEISEEQKAEYQRKSEALKERFINRQKSK-NESVVSLIDDED-----DNENDRQLVVSA 345
ABR51685.1      TQEISEEQKAEYQRKSEALKERFINRQKSK-NESVVSLIDDED-----DNENDRQLVVSA 345
ADC36978.1      TQEISEEQKAEYQRKSEALKERFINRQKSK-NESVVSLIDDED-----DNENDRQLVVSA 345
CCJ10583.1      TQEISEEQKAEYQRKSEALKERFINRQKSK-NESVVSLIDDED-----DNENDRQLVVSA 345
ADI97344.1      SKEVSEQQKADYERKAKERKERFLDRQKSK-KEPVVSLEYDFEHKQRFNNANEKQLVVSA 350
ABD20992.1      SQEVSEQQKAEYDKRAEERKARFLDNQKIK-KTPVVSLEYDFEHKQRIDNENDKKLVVSA 353
CBI48742.1      SQEVSEQQKAEYDKRAEERKARFLDNQKIK-KTPVVSLEYDFEHKQRIDNENDKKLVVSA 353
AAW36412.1      SQEVSEQQKAEYDKRAEERKARFLDNQKIK-KTPVVSLEYDFEHKQRIDNENDKKLVVSA 353
ELP41199.1      SQEVSEQQKAEYDKRAEERKARFLDNQKIK-KTPVVSLEYDFEHKQRIDNENDKKLVVSA 353
AGO29164.1      SQEVTAEQKADYEKRAEERKARFLDRQKSK-KEPVVSLEYDFEHKQRVDNANDKQLVVSA 355
ADQ77852.1      SQEVSEQQKADYERKAEERKARFLDKQKNK-KTPVVSLEYDFEHKQRVDNENDKQLVVSE 347
AIO20460.1      SQEVSEQQKADYERKAEERKARFLDKQKNK-KTPVVSLEYDFEHKQRVDNENDKQLVVSE 347
AEW64854.1      SKEVTAEQKAEYEKRAEERKARFLDRQKSK-KEPVVSLEYDFEHKQRFNNANDKQLVVSA 359
AGU54602.1      SQEVSEKQKADYERKAEERKARFLDKQKNK-QTPVVSLEYDFEHKQRVDNANDKQLVVSA 351
ADL22691.1      SQEVSEQQKAEYEKRAEERKARFLDKQKNK-QTPLMSLEYDFEHKQRVDNENDKQLVVSA 349
                 .*:: :***:*::: :  * :*: .:*   .  ::**  * :        *:::**:*

AMV79382.1      PSKKPTTPTTYTETTTQAPMPTVERQTQQQIIYNAPKQLAGLNGESHDFTTTHQSPTTSN 407
AFH69100.1      PTNKPTTLPTYTETTTQVPMPTVERQTQQQIVYKAPKPLAGLNGESHDFTTTHQSPTTSN 409
AMV84568.1      PTKKPTSPTTYTETTTQVPMPTVERQTQQQIIYNAPKQLAGLNGESHDFTTTHQSPTTSN 407
CRL33493.1      PSKKPTTPTTYTETTTQVPMPTVERQTQQQIIYNAPKQLAGLNGESHDFTTTHQSPTTSN 404
ANI73770.1      PTKKPTTPTTYTETTTQVPMPTVERQTQQQIVYKTPKPLARLNGESHDFTTTHQSPTTSN 407
EJE56571.1      PTKKPTTPTTYTETTTQVPMPTVERQTQQQIIYNAPKQLAGLNGESHDFTTTHQSPTTSN 404
CAI80433.1      PTKKPTSPTSYTETTTQVPMPTVERQTQQQIIYNTPKQLAGLNGESHDFTTTHQSSTTSN 404
CCG15432.1      PSKKPTTPPTYTETTTQVPMPTVERQTQQQIIYNAPKQLAGLNGESHDFTTTHQSPTTSN 407
AEV77847.2      PTKKPTSPTTYTETMTQVPMPTVERQAHQQIVYKAPKQLAGLNGESHDFTTTHQSPTTSN 407
ALQ99048.1      PTKKPTSPTTYTETMTQVPMPTVERQAHQQIVYKAPKQLAGLNGESHDFTTTHQSPTTSN 407
BAF77691.1      PSKKPTTPTTYTETTTQVPMPTVERQTQQQIVYKTPKPLAGLNGESHDFTTTHQSPTTSN 405
AQD18904.1      PSKKPTTPTTYTETTTQVPMPTVERQTQQQIVYKTPKPLAGLNGESHDFTTTHQSPTTSN 405
ABR51685.1      PSKKPTTPTTYTETTTQVPMPTVERQTQQQIVYKTPKPLAGLNGESHDFTTTHQSPTTSN 405
ADC36978.1      PSKKPTTPTTYTETTTQVPMPTVERQTQQQIVYKTPKPLAGLNGESHDFTTTHQSPTTSN 405
CCJ10583.1      PSKKPTTPTTYTETTTQVPMPTVERQTQQQIVYKTPKPLAGLNGESHDFTTTHQSPTTSN 405
ADI97344.1      PTKKPT-PPTYTETTTQVPMPTVERQTQEQIVYKAPKQLAGLNGESHDFSTTHQTPTTSN 409
ABD20992.1      PTKKPTSPTTYTETTTQVPMPTVERQTQQQIIYNAPKQLAGLNGESHDFTTTHQSPTTSN 413
CBI48742.1      PTKKPTSPTTYTETTTQVPMPTVERQTQQQIIYNAPKQLAGLNGESHDFTTTHQSPTTSN 413
AAW36412.1      PTKKPTSPTTYTETTTQVPMPTVERQTQQQIIYNAPKQLAGLNGESHDFTTTHQSPTTSN 413
```

```
ELP41199.1      PTKKPTSPTTYTETTTQVPMPTVERQTQQQIIYNAPKQLAGLNGESHDFTTTHQSPTTSN 413
AGO29164.1      PTKKPTLP-TYTETTTQVPMPTVERQTQEQIVYKAPKQLAGLNGESHNFSTTHQTPITSN 414
ADQ77852.1      PSKKPTTPPTYTETTTQLPMPTVERQTQQQIVYKAPKPLAGLNGESHDFTTTHQSPTTSN 407
AIO20460.1      PSKKPTTPPTYTETTTQLPMPTVERQTQQQIVYKAPKPLAGLNGESHDFTTTHQSPTTSN 407
AEW64854.1      PTKKPTTPTTYTETTTQVPMPTVERQTQQQIVYKTPKPLVGLNGESHDFTTTHQSPTTSN 419
AGU54602.1      PTKKPTSPTSYTETTTQVPMPTVERQTQQQIVYKAPKQLAGLNGESHDFTTTHQSPTTSN 411
ADL22691.1      PTKKPTPPPTYTETTTQVPMPAVERQAQQQIVYKTPKPLAGLNGESHDFTTTHQSPTTSN 409
                *::***   :**** ** ***:****:::**:*::** *. ******:*:****:  ***

AMV79382.1      HTHNHLIEFEETSALPGRKSGSLVGISQIDSSHLTEREKRVIKREHVREAQKLVDNYKDT 467
AFH69100.1      HTHNHLIEIEETSALPSRKTGSLVGLSQIDSSHLTEREKRVIKREHVREAQKLVDNYKDT 469
AMV84568.1      HTHNNVVEFEETSALPGRKSGSLVGISQIDSSHLTEREKRVIKREHVREAQKLVDNYKDT 467
CRL33493.1      HTHSNLIEFEETSALPGRKSGSLVGISQIDSSHLTEREKRVIKREHVREAQKLVDNYKDT 464
ANI73770.1      HTHNNVVEFEETSALPGRKSGSLVGISQIDSSHLTEREKRVIKREHVREAQKLVDNYKDT 467
EJE56571.1      HTHNNVVKFEETSALPGRKSGSLVGISQIDSSHLTEREKRVIKREHVREAQKLVDNYKDT 464
CAI80433.1      HTHNNVVEFEETSALPGRKSGSLVGISQIDSSHLTEREKRVIKREHVREAQKLVDNYKDT 464
CCG15432.1      HTHNHLIEFEETSALPGRKSGSLVGISQIDSSHLTEREKRVIKREHVREAQKLVDNYKDT 467
AEV77847.2      HTHNNVVEFEETSALPGRKSGSLVGISQIDSSHLTEREKRVIKREHVREAQKLVDNYKDT 467
ALQ99048.1      HTHNNVVEFEETSALPGRKSGSLVGISQIDSSHLTEREKRVIKREHVREAQKLVDNYKDT 467
BAF77691.1      HTHNNVVEFEETSALPGRKSGSLVGISQIDSSHLTEREKRVIKREHVREAQKLVDNYKDT 465
AQD18904.1      HTHNNVVEFEETSALPGRKSGSLVGISQIDSSHLTEREKRVIKREHVREAQKLVDNYKDT 465
ABR51685.1      HTHNNVVEFEETSALPGRKSGSLVGISQIDSSHLTEREKRVIKREHVREAQKLVDNYKDT 465
ADC36978.1      HTHNNVVEFEETSALPGRKSGSLVGISQIDSSHLTEREKRVIKREHVREAQKLVDNYKDT 465
CCJ10583.1      HTHNNVVEFEETSALPGRKSGSLVGISQIDSSHLTEREKRVIKREHVREAQKLVDNYKDT 465
ADI97344.1      HTHNNVVEFEETSALPGRKTGSLVGLSQIDSSHLTEREKRVIKREHVREAQKLVENYKDT 469
ABD20992.1      HTHNNVVEFEETSALPGRKSGSLVGISQIDSSHLTEREKRVIKREHVREAQKLVDNYKDT 473
CBI48742.1      HTHNNVVEFEETSALPGRKSGSLVGISQIDSSHLTEREKRVIKREHVREAQKLVDNYKDT 473
AAW36412.1      HTHNNVVEFEETSALPGRKSGSLVGISQIDSSHLTEREKRVIKREHVREAQKLVDNYKDT 473
ELP41199.1      HTHNNVVEFEETSALPGRKSGSLVGISQIDSSHLTEREKRVIKREHVREAQKLVDNYKDT 473
AGO29164.1      HTHNNVVEFEETSALPGRKTGSLVGLSQIDSSHLTEREKRVIKREHVREAQKLVENYKDT 474
ADQ77852.1      HTHNHLIEIEETSALPGRKTGSLVGLSQIDSSHLTEREKRVIKREHVREAQKLVDNYKDT 467
AIO20460.1      HTHNHLIEIEETSALPGRKTGSLVGLSQIDSSHLTEREKRVIKREHVREAQKLVDNYKDT 467
AEW64854.1      HTHNNVVEFEETSALPGRKSGSLVGISQIDSSHLTEREKRVIKREHVREAQKLVDNYKDT 479
AGU54602.1      NTHNHLIEFEETSALPGRKSGSLVGISQIDSSHLTEREKRVIKREHVREAQKLVDNYKDT 471
ADL22691.1      HTHNNVVEFEETSALPGRKSGSLVGLSQIDSSHLTEREKRVIKREHVREAQKLVDNYKDT 469
                :**.:::::*******.**:*****:****************************:*****

AMV79382.1      HSYKDRLNAQQKVNTLSEGHQKRFNKQINKVYNGK         502
AFH69100.1      HSYKDRLNAQQKVNTLSAGHQKRFNKQINKVYNGK         504
AMV84568.1      HSYKDRLNAQQKVNTLSEGHQKRFNKQIDKVYNGK         502
CRL33493.1      HSYKDRLNAQQKVNTLSEGHQKRFNKQINKVYNGK         499
ANI73770.1      HSYKDRLNAQQKVNTLSEGHQKRFNKQIDKVYNGK         502
EJE56571.1      HSYKDRLNAQQKVNTLSEGHQKRFNKQINKVYNGK         499
CAI80433.1      HSYKDRLNAQQKVNTLSEGHQKRFNKQINKVYNGK         499
CCG15432.1      HSYKDRLNAQQKVNTLSEGHQKRFNKQINKVYNGK         502
AEV77847.2      HSYKDRLNAQQKVNTLSEGHQKRFNKQIDKVYNGK         502
ALQ99048.1      HSYKDRLNAQQKVNTLSEGHQKRFNKQIDKVYNGK         502
BAF77691.1      HSYKDRLNAQQKVNTLSEGHQKRFNKQINKVYNGK         500
AQD18904.1      HSYKDRLNAQQKVNTLSEGHQKRFNKQINKVYNGK         500
ABR51685.1      HSYKDRLNAQQKVNTLSEGHQKRFNKQINKVYNGK         500
ADC36978.1      HSYKDRLNAQQKVNTLSEGHQKRFNKQINKVYNGK         500
CCJ10583.1      HSYKDRLNAQQKVNTLSEGHQKRFNKQINKVYNGK         500
ADI97344.1      HSYKDRLNAQQKVNTLSSGHQKRFNKQINKVYNGK         504
ABD20992.1      HSYKDRINAQQKVNTLSEGHQKRFNKQINKVYNGK         508
CBI48742.1      HSYKDRINAQQKVNTLSEGHQKRFNKQINKVYNGK         508
AAW36412.1      HSYKDRINAQQKVNTLSEGHQKRFNKQINKVYNGK         508
ELP41199.1      HSYKDRINAQQKVNTLSEGHQKRFNKQINKVYNGK         508
AGO29164.1      HSYKDRLNAQQKVNTLSEGHQKRFNKQINKVYNGK         509
ADQ77852.1      HSYKDRLNAQQKVNTLSAGHQKRFNKQINKVYNGK         502
AIO20460.1      HSYKDRLNAQQKVNTLSAGHQKRFNKQINKVYNGK         502
AEW64854.1      HSYRDRLNAQQKVNTLSEGHQKRFNKQINKVYNGK         514
AGU54602.1      HSYKDRLNAQQKVNTLSEGHQKRFNKQIDKVYNGK         506
ADL22691.1      HSYKDRLNAQQKVNTLSEGHQKRFNKQINKVYNGK         504
```

***:**:********** *********:******
