# Supplementary material for: vhp Is a Fibrinogen-Binding Protein Related to vWbp in Staphylococcus aureus
Source: mBio. 2021 Aug 3;12(4):e01167-21. doi: 10.1128/mBio.01167-21 (PMC8406236; doi:10.1128/mBio.01167-21)
Supplement: FIG S2 [file mbio.01167-21-sf002.pdf]

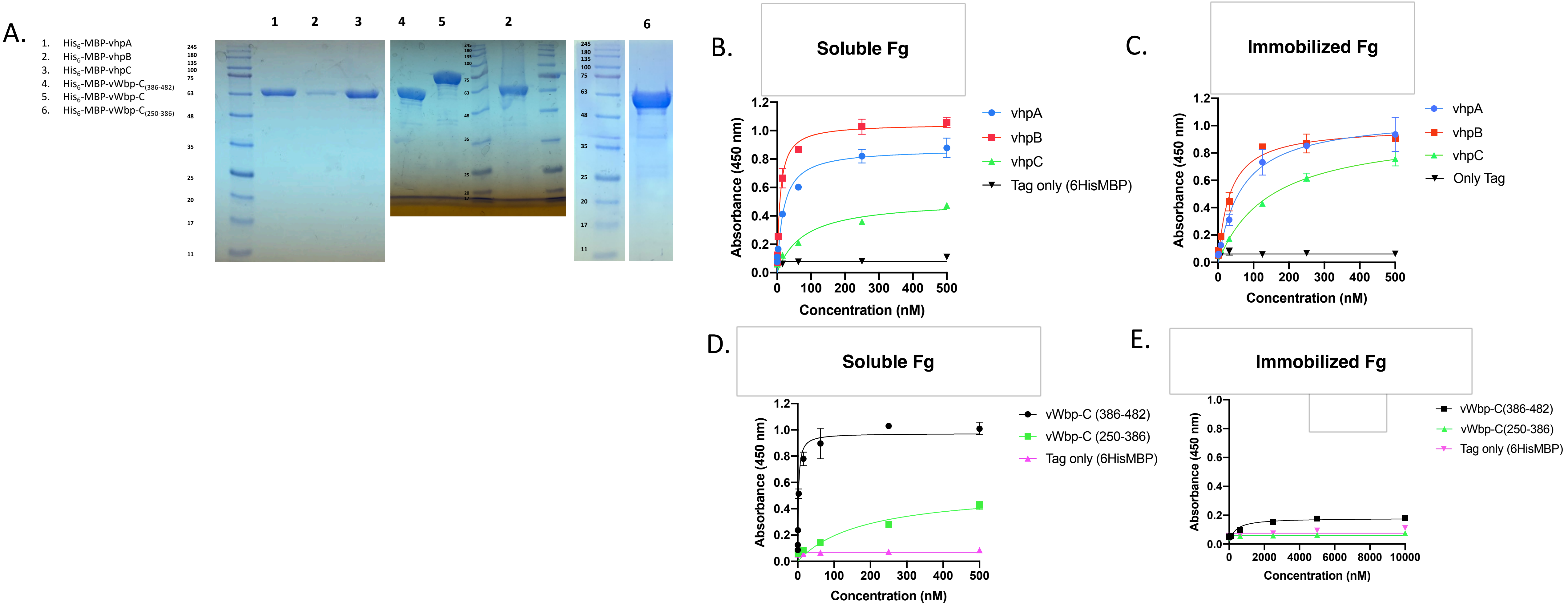

Fig S2. Both vWbp-C$_{(386\text{-}482)}$ and vhp bind Fg. (A) SDS-PAGE gel of vhp isoforms and vWbp-C constructs. (B) ELISA binding of soluble Fg to immobilized vhp isoforms (0.5 µg/well). (C) ELISA binding of vhp isoforms to immobilized Fg (0.5 µg/well). (D) ELISA binding of soluble Fg to immobilized vWbp-C constructs (0.5 µg/well). (E) ELISA binding of vWbp-C constructs binding to immobilized Fg (0.5 µg/well). Error bars, standard error of the mean (SEM). The graphs are representative of three independent experiments.
